# *B*. *abortus* RNA is the component involved in the down-modulation of MHC-I expression on human monocytes via TLR8 and the EGFR pathway

**DOI:** 10.1371/journal.ppat.1006527

**Published:** 2017-08-02

**Authors:** M. Ayelén Milillo, Lis N. Velásquez, Aldana Trotta, M. Victoria Delpino, Fábio V. Marinho, Luciana Balboa, Mónica Vermeulen, Sonia L. Espindola, Nahuel Rodriguez-Rodrigues, Gabriela C. Fernández, Sergio Costa Oliveira, Guillermo H. Giambartolomei, Paula Barrionuevo

**Affiliations:** 1 Instituto de Medicina Experimental (CONICET-Academia Nacional de Medicina), Buenos Aires, Argentina; 2 Instituto de Inmunología, Genética y Metabolismo (CONICET-UBA), Laboratorio de Inmunogenética, Buenos Aires, Argentina; 3 Departamento de Bioquímica e Imunologia, Universidade Federal de Minas Gerais, Belo Horizonte, Brazil; 4 Instituto de Investigaciones en Ingeniería Genética y Biología Molecular (INGEBI), CONICET, Buenos Aires, Argentina; McMaster University, CANADA

## Abstract

Despite eliciting a potent CD8^+^ T cell response, *Brucella abortus* is able to persist and establish a chronic infection inside its host. We have previously reported that the infection of human monocytes/macrophages with *B*. *abortus* inhibits the IFN-γ-induced MHC-I cell surface expression down-modulating cytotoxic CD8^+^ T cell responses. MHC-I down-modulation depends on bacterial viability and results from the capacity of *B*. *abortus* to retain the MHC-I molecules within the Golgi apparatus. Furthermore, we recently demonstrated that epidermal growth factor receptor (EGFR) pathway is involved in this phenomenon and that this is an early event during infection. However, the components and mechanisms whereby *B*. *abortus* is able to down-modulate MHC-I remained to be elucidated. In this study we demonstrated that the down-modulation of MHC-I expression is not mediated by well-known *Brucella* virulence factors but instead by *B*. *abortus* RNA, a PAMP associated to viability (*vita*-PAMP). Surprisingly, completely degraded RNA was also able to inhibit MHC-I expression to the same extent as intact RNA. Accordingly, *B*. *abortus* RNA and its degradation products were able to mimic the MHC-I intracellular retention within the Golgi apparatus observed upon infection. We further demonstrated that TLR8, a single-stranded RNA and RNA degradation products sensor, was involved in MHC-I inhibition. On the other hand, neutralization of the EGFR reversed the MHC-I inhibition, suggesting a connection between the TLR8 and EGFR pathways. Finally, *B*. *abortus* RNA-treated macrophages display diminished capacity of antigen presentation to CD8^+^ T cells. Overall, our results indicate that the *vita*-PAMP RNA as well as its degradation products constitute novel virulence factors whereby *B*. *abortus*, by a TLR8-dependent mechanism and through the EGFR pathway, inhibits the IFN-γ-induced MHC-I surface expression on human monocytes/macrophages. Thus, bacteria can hide within infected cells and avoid the immunological surveillance of cytotoxic CD8^+^ T cells.

## Author summary

*Brucella abortus* is one of the intracellular bacterial species that cause brucellosis, a zoonotic worldwide disease. An intricate enigma of *Brucella* immunity is its long-term persistence inside host despite a vigorous and specific immune response. Our study describes a novel immune evasion strategy exploited by *B*. *abortus*: the down-modulation of the IFN-γ-induced expression of the molecules responsible for antigen presentation to CD8^+^ T cells on the surface of monocytes, Major Histocompatibility Complex Class I (MHC-I) molecules. We found that the bacterial component responsible for this phenomenon is its RNA, a component associated with bacterial viability itself. Specifically, we demonstrated that not only does intact RNA down-modulate MHC-I but also the RNA degradation products. Bacterial RNA is sensed by pattern recognition receptors (PRRs), among which TLRs family has gained more attention. In this study, we demonstrated that the receptor involved in this phenomenon is TLR8, a single-stranded RNA and RNA degradation products sensor. By means of this mechanism, *Brucella* impairs antigen presentation to CD8^+^ T cells, hiding within infected cells and avoiding the immunological surveillance of cytotoxic cells. On balance, these results provide new evidence to understand how *B*. *abortus* can survive inside the host and persist chronically.

## Introduction

Host control of brucellosis requires a set of cells and components of the immune system which together promote a complex response against *Brucella* spp. [1]. Yet, from the many defensive resources that adaptive immunity brings into play, cytotoxic CD8^+^ T cells are determinant to restrain *Brucella* infection. The importance of these cells resides in their capacity to eliminate *Brucella*-infected target cells [2, 3]. Previous studies in humans, mice and bovines have shown that specific CD8^+^ T cells are developed during *Brucella* infection [1, 4], confirming the ability of *Brucella*-infected macrophages to present bacterial antigens on MHC-I molecules and activate cytotoxic CD8^+^ T cell responses. Despite this immune response, *Brucella* is able to persist inside these cells establishing a chronic infection. Therefore, as a successful chronic and persistent pathogen, *Brucella* must own an effective strategy to subvert the challenge of highly outfitted CD8^+^ T cells. We have previously reported that infection of human monocytes/macrophages with *B*. *abortus* inhibits the IFN-γ-induced MHC-I cell surface expression. As a consequence, *B*. *abortus*-infected macrophages display diminished capacity of antigen presentation to CD8^+^ T cells [5]. MHC-I down-modulation results from the capacity of *B*. *abortus* to induce the retention of these molecules within the Golgi apparatus [5]. However, the components of *B*. *abortus* involved in this phenomenon remained unknown.

Interestingly, *B*. *abortus*-mediated MHC-I down-modulation is dependent on bacterial viability as was demonstrated by the incapacity of heat-killed bacteria to inhibit the expression of such molecules [5]. Furthermore, we have recently reported that two *B*. *abortus* mutant strains devoid of key virulence factors, *B*. *abortus* RB51 (a rough LPS mutant) and *B*. *abortus virB10*^-^ (a VirB type IV secretion system mutant), are capable of inhibiting the IFN-γ-induced MHC-I surface expression to the same extent as wild-type *B*. *abortus* [6]. These *B*. *abortus* mutant strains are unable to persist inside human monocytes for a long period despite their preserved capacity of infecting cells [7–9]. Consistent with this, we observed that the phenomenon of MHC-I inhibition is triggered at early time points and can be observed at 8 h post-infection. At 24 h and 48 h post-infection it became even stronger [6]. Overall these results led us to think that the components involved in the inhibition of IFN-γ-induced MHC-I surface expression should be associated with bacterial viability. In turn, our results with the mutant strains gave us the idea that these bacterial components should be expressed early during infection.

It has been recently demonstrated that the immune system is capable of sensing the most essential characteristic of microbial infectivity, microbial viability itself [10]. The immune system can thus detect pathogen-associated molecular patterns (PAMPs) which are present in live bacteria but rapidly eliminated when bacteria lose their viability [10]. These PAMPs are lost since they are intimately linked to the metabolic activity and replicative capacity of the microorganisms. In order to differentiate them from traditional PAMPs, structural components that are preserved after loss of bacterial viability (such as LPS, lipoproteins and DNA, among others), this special class of PAMPs were named viability-associated PAMPs (*vita*-PAMPs), among which prokaryotic RNA is included [10, 11].

Recognition of nucleic acids in general and RNA in particular by receptors of the innate immune system is a complicated and interesting field of investigation. The immune system must discriminate between ‘self’ (host) and ‘foreign’ (invading pathogen) nucleic acids [12]. This principle is based on three criteria: the availability of nucleic acid ligands, the localization of such nucleic acids and their structure (characterized by sequence motifs, conformation and chemical modification). In most cases, a combination of these aspects contributes to the proper recognition of foreign nucleic acids and the induction of adequate immune responses [12]. Most of the receptors involved in the immune sensing of nucleic acids have been identified. Among them, the TLRs located in endosomes/phagolysosomes are the most studied: TLR9 senses CpG DNA motifs; TLR3 and TLR7 are capable of recognizing double-stranded and single-stranded RNA respectively and TLR8 is not only able to recognize single-stranded RNA but it has been recently described as a RNA degradation products sensor as well [13, 14].

Taking our previous results into account, we hypothesized that the components of *B*. *abortus* involved in the inhibition of MHC-I could be *vita*-PAMPs such as *B*. *abortus* RNA, since they are found exclusively in live bacteria and are actively expressed during early stages of infection. Thus, the aim of this study was to characterize the components, signaling pathways and mechanisms implicated in MHC-I down-modulation. Overall, our results indicate that the *vita*-PAMP RNA as well as its degradation products constitute novel virulence factors whereby *B*. *abortus*, by a TLR8-dependent mechanism and through the EGFR pathway, inhibits the IFN-γ-induced MHC-I surface expression on human monocytes/macrophages.

## Results

### The inhibition of MHC-I surface expression mediated by *B*. *abortus* is dependent on bacterial viability but independent of its clue virulence factors

**Fig 1:**
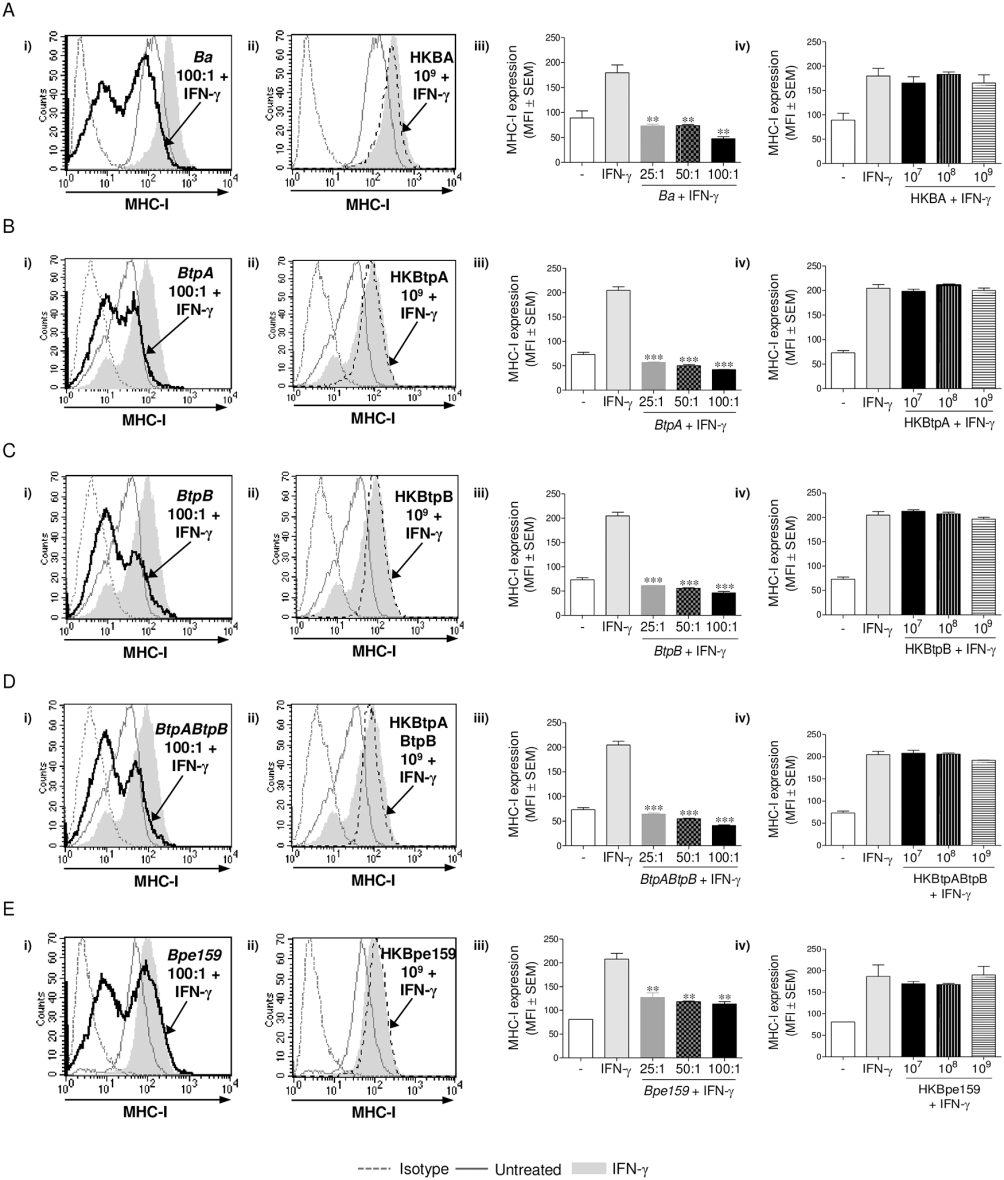
Only viable *B*. *abortus*, independently of its virulence factors, is able to inhibit MHC-I expression. (A-E, Panels i and iii) THP-1 cells were infected with *B*. *abortus* WT (A), *btpA* (B), *btpB* (C), *btpAbtpB* (D) and *Bpe159* (E) at different MOI in the presence of IFN-γ for 2 h, washed and cultured in the presence of IFN-γ for 48 h. (A-E, Panels ii and iv) At the same time, heat-killed (HK) bacteria were used to treat THP-1 cells in the presence of IFN-γ for 48 h. MHC-I expression was assessed by flow cytometry. Bars represent the arithmetic means ± SEM of five experiments. MFI, mean fluorescence intensity. **P<0.01; ***P<0.001 *vs*. IFN-γ-treated.

**Fig 2:**
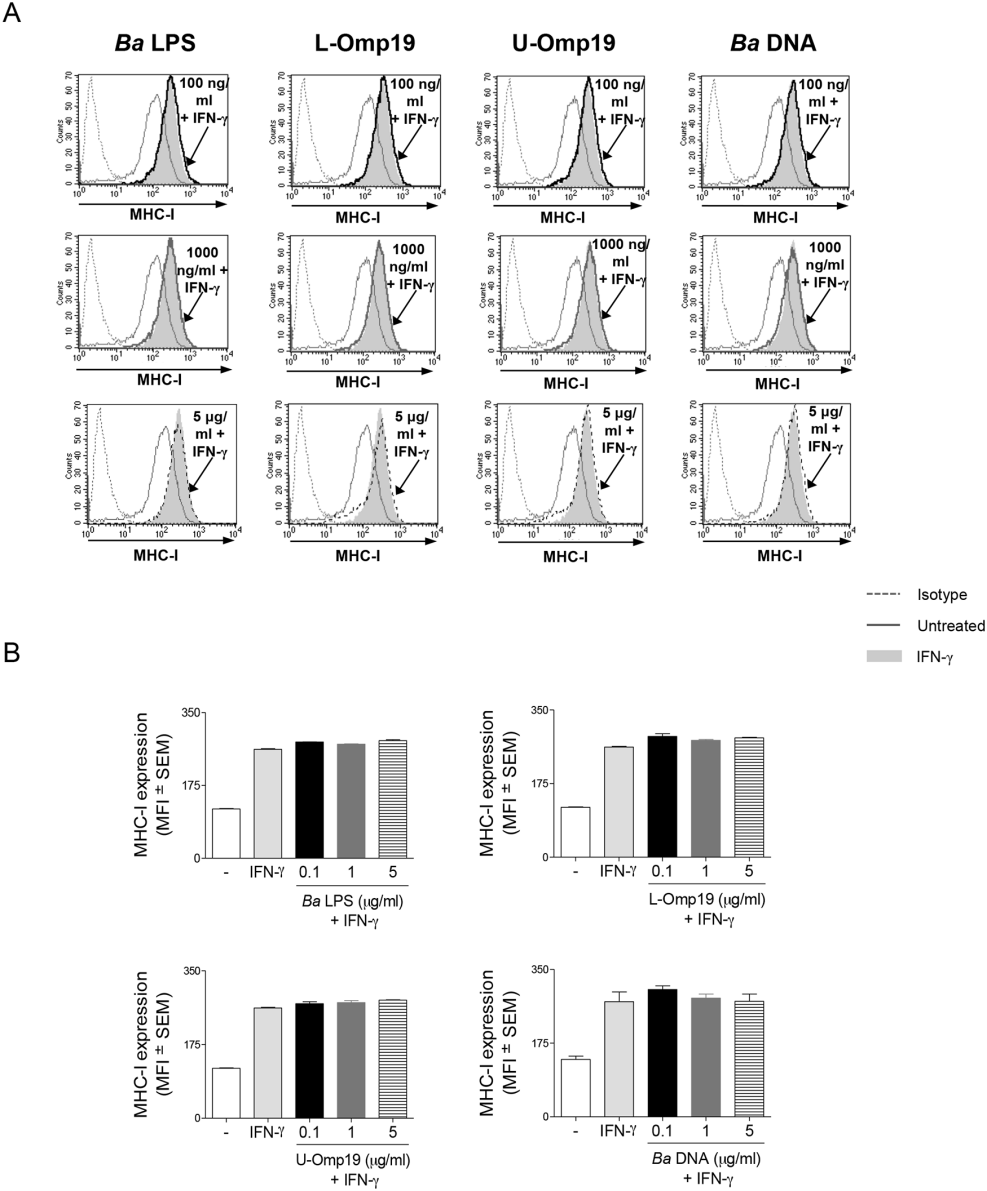
*B*. *abortus* structural components do not down-modulate the IFN-γ-induced MHC-I expression on THP-1 cells. (A and B) THP-1 cells were treated with different doses of *B*. *abortus* LPS, L-Omp19, U-Omp19 or DNA in the presence of IFN-γ for 48 h. MHC-I expression was assessed by flow cytometry. Bars indicate the arithmetic means ± SEM of five independent experiments. MFI, mean fluorescence intensity.

Our previous results had demonstrated that *B*. *abortus*-mediated MHC-I inhibition is dependent on bacterial viability [5]. On the other hand, we have recently reported that *B*. *abortus* rough LPS mutant RB51 and a mutant in the *B*. *abortus* type IV secretion system VirB, two mutant strains in key virulence factors, are capable of inhibiting the IFN-γ-induced MHC-I surface expression to the same extent as wild-type *B*. *abortus* [6]. These results led us to think that human monocytes/macrophages could be able to detect a component associated with bacterial viability independently of its virulence factors. In order to address this hypothesis, we used different *B*. *abortus* mutant strains on key virulence factors and evaluated whether their live and heat-killed (HK) forms could inhibit the IFN-γ-induced MHC-I surface expression on THP-1 cells. The mutant strains used were: RB51 (rough LPS mutant), *virB10*^-^ (mutant in VirB type IV secretion system), *btpA*, *btpB* single mutants and a *btpAbtpB* double mutant (mutants of TIR domain-containing proteins which interfere with TLRs signaling pathways), and *Bpe159* (mutant in *B*. *abortus* putative effector protein BPE159, which is secreted into the host cytosol independently of the VirB secretion system [15]). Confirming and extending our previous results, *B*. *abortus* S2308 (wild type, WT) and all mutant strains studied were able to diminish the IFN-γ-induced MHC-I surface expression in a dose-dependent manner after 48 h. However, this phenomenon occurred exclusively when bacteria were alive (Fig 1A–1E and S1 Fig, Panels i and iii). The heat-killed forms of these bacteria lost the capacity of inhibiting MHC-I, even at the highest concentration used (1 x 10^9^ bacteria/ml) (Fig 1A–1E and S1 Fig, Panels ii and iv). These results confirm that the inhibition of MHC-I surface expression is dependent on *B*. *abortus* viability but independent of the studied virulence factors. In addition, these results suggest that MHC-I inhibition is not caused by *B*. *abortus* structural components, which are conserved in heat-killed bacteria. To corroborate our results, we next studied the effect of different structural components of *B*. *abortus* on MHC-I surface expression, such as: *B*. *abortus* lipopolysaccharide (*Ba* LPS); its outer membrane protein 19 (Omp19), a prototypical lipoprotein of *B*. *abortus*, on its lipidated (L-Omp19) and unlipidated (U-Omp19) versions and *B*. *abortus* DNA (*Ba* DNA). None of the evaluated structural components was able to inhibit the IFN-γ-induced MHC-I surface expression (Fig 2A and 2B). Overall, these results confirm that *B*. *abortus*-mediated inhibition of MHC-I surface expression requires bacterial viability regardless of the presence of more specialized factors that regulate microbial virulence. In addition, they show that the bacterial component involved in this phenomenon seems to be associated with bacterial viability.

### *B*. *abortus* RNA is the *vita*-PAMP involved in the inhibition of MHC-I expression

**Fig 3:**
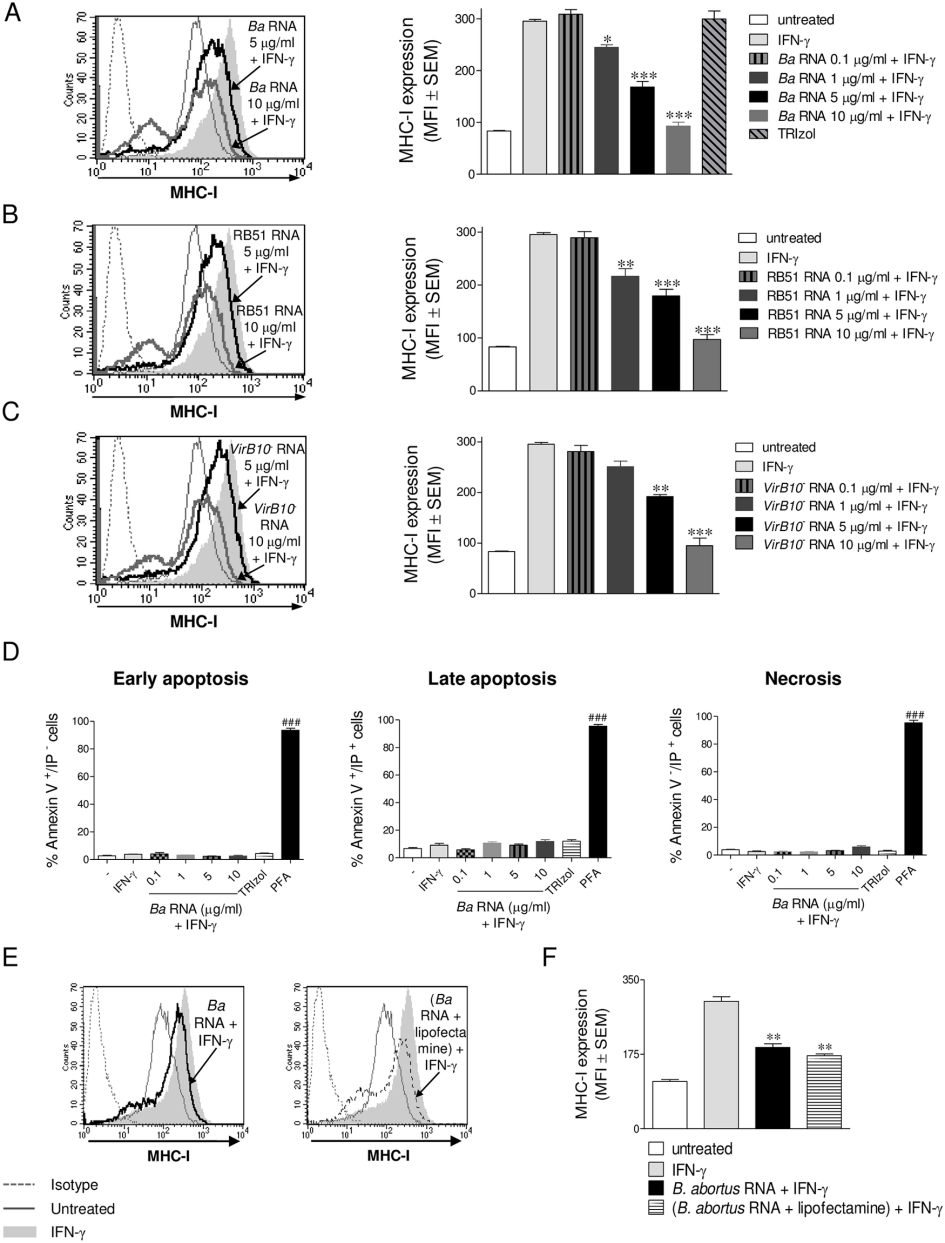
*B*. *abortus* RNA inhibits MHC-I expression and this does not involve loss of cell viability. (A) THP-1 cells were treated with different doses of *B*. *abortus* WT RNA in the presence of IFN-γ for 48 h. THP-1 cells treated with TRIzol extracted products in the absence of bacteria were used as a control. (B and C) THP-1 cells were treated with different doses of RB51 (B) and *virB10*^-^ (C) RNAs in the presence of IFN-γ for 48 h. MHC-I expression was assessed by flow cytometry. (D) THP-1 cells treated with different doses of *B*. *abortus* WT RNA in the presence of IFN-γ for 48 h were stained with Annexin V-FITC and Propidium Iodide (PI) and then analyzed for early Apoptosis (Annexin V^+^/PI^-^), late apoptosis (Annexin V^+^/PI^+^) and necrosis (Annexin V^-^/PI^+^). Cells treated with Paraformaldehyde (PFA) were used as a positive control. (E and F) THP-1 cells were transfected with *B*. *abortus* WT RNA with lipofectamine or treated with *B*. *abortus* WT RNA in the presence of IFN-γ for 48 h. MHC-I was assessed by flow cytometry. Bars represent the arithmetic means ± SEM of five experiments. MFI, mean fluorescence intensity. *P<0.05; **P<0.01; ***P<0.001 *vs*. IFN-γ-treated. ^###^*P*<0.001 *vs*. untreated.

Prokaryotic RNA has been recently characterized as a special class of viability-associated PAMP (*vita*-PAMP), as it is present only in viable bacteria [10]. To investigate whether *B*. *abortus* RNA was the component involved in the inhibition of MHC-I surface expression on human monocytes, we used *B*. *abortus* WT RNA to stimulate THP-1 cells in the presence of IFN-γ for 48 h at different concentrations. The expression of MHC-I was then evaluated by flow cytometry. *B*. *abortus* WT RNA significantly down-regulated the IFN-γ-induced surface expression of MHC-I molecules in a dose-dependent manner (Fig 3A), mimicking what was previously observed with viable *B*. *abortus*. Given that certain phenol traces could still be present in the purified RNA, we performed the RNA extraction in the absence of bacteria and used it as control (TRIzol bar). This treatment was not able to down-modulate MHC-I (Fig 3A). Moreover, RNA purified with a method other than TRIzol was equally able to inhibit MHC-I expression on THP-1 cells (S2 Fig). In turn, RNA purified from the mutant strains RB51 and *virB10* was also able to inhibit the IFN-γ-induced MHC-I surface expression on THP-1 cells to the same extent as *B*. *abortus* WT RNA (Fig 3B and 3C). This inhibition was not due to a loss of cell viability in *B*. *abortus* RNA stimulated cultures, since MHC-I inhibition was observed gating only on viable cells (7-AAD negative cells). Furthermore, *B*. *abortus* RNA treatment did not induce early and late apoptosis or necrosis as determined by the Annexin V assay, even at the highest evaluated concentration (10 μg/ml) (Fig 3D). On the contrary, high levels of early and late apoptosis or necrosis were obtained on cells treated with the positive control paraformaldehyde (PFA).

In agreement with the inability of heat-killed *B*. *abortus* (HKBA) to inhibit MHC-I surface expression, we determined by gel electrophoresis that HKBA lacked RNA and that the products recovered from the HKBA RNA extraction process were unable to inhibit MHC-I surface expression (S3 Fig). Moreover, *B*. *abortus* RNA was able to complement the absence of this molecule in HKBA, making it capable of down-modulating MHC-I expression on human monocytes (S4 Fig). In another set of experiments, THP-1 cells were stimulated with *B*. *abortus* RNA alone or introduced into the cell by transfection with lipofectamine reagent. Stimulation with *B*. *abortus* RNA, independently of the procedure, was able to inhibit MHC-I expression suggesting that RNA without transfection could gain access to endosomal sensors (3E and F). Confirming these results, the endocytosis inhibitor Nystatin was able to reverse MHC-I inhibition mediated by stimulation with *B*. *abortus* RNA (S5 Fig). On the other hand, other prokaryotic RNAs (from *Bacillus cereus*, *Salmonella typhimurium*, *Escherichia coli* and *Klebsiella pneumonia*) were able to inhibit MHC-I surface expression. On the contrary, eukaryotic RNA (from peripheral blood mononuclear cells, PBMCs) was unable to inhibit MHC-I surface expression, even at the highest concentration utilized (S6 Fig). Overall, these results indicate that RNA is a component associated with bacterial viability which is employed by *B*. *abortus* to inhibit the IFN-γ-induced surface expression of MHC-I molecules on human monocytes. More importantly, this is not an exclusive phenomenon of *B*. *abortus* RNA as it could be extended to other prokaryotic although not to eukaryotic RNAs.

### *B*. *abortus* RNA degradation products are also able to inhibit the IFN-γ-induced expression of MHC-I

**Fig 4:**
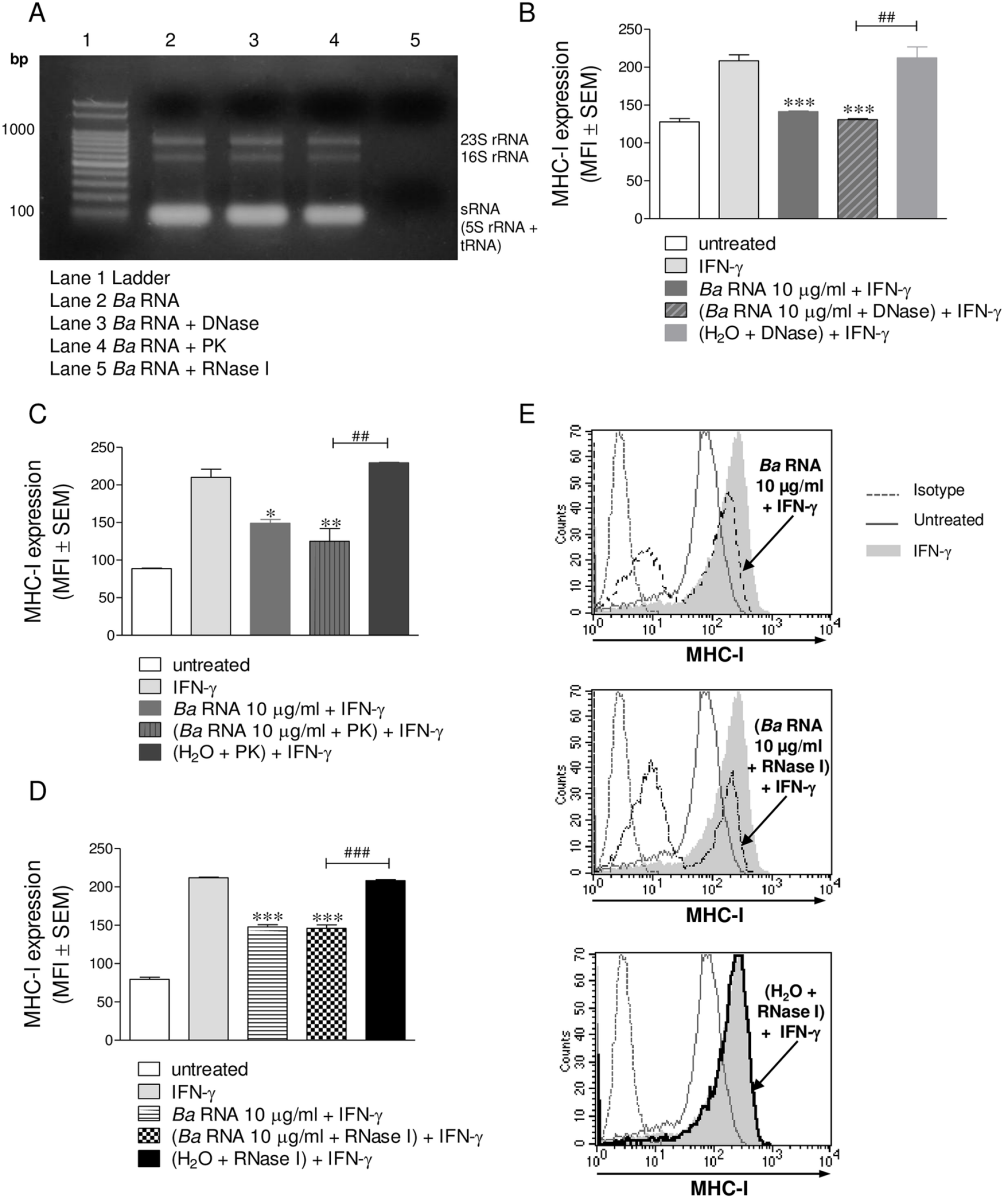
*B*. *abortus* RNA degradation products are also capable of inhibiting the IFN-γ-induced expression of MHC-I. (A) RNA from *B abortus* was purified and treated with DNase, Proteinase K (PK) or *E*. *coli* RNase I. Each treatment was visualized by 1% agarose gel electrophoresis. (B and C) THP-1 cells were stimulated with DNase (B) or PK (C)–treated *B*. *abortus* RNA in the presence of IFN-γ for 48 h. Cells treated with DNase or PK alone were used as negative controls. Cells treated with *B*. *abortus* RNA were used as positive controls. (D and E) THP-1 cells were treated with RNase I-treated *B*. *abortus* RNA in the presence of IFN-γ for 48 h. Cells treated only with RNase I were used as negative controls. Cells treated with *B*. *abortus* RNA were used as positive controls. MHC-I was assessed by flow cytometry. Bars represent the arithmetic means ± SEM of five experiments. MFI, mean fluorescence intensity. *P<0.05; **P<0.01; ***P<0.001 *vs*. IFN-γ-treated. ^##^*P*<0.01; ^###^*P*<0.001 *vs*. negative controls.

As traces of DNA and proteins could contaminate the RNA fractions, we decided to further purify our preparations of *B*. *abortus* RNA by eliminating either residual DNA or proteins. For this, *B*. *abortus* RNA fractions were digested with a DNase or a proteinase (Proteinase K; PK). After that, we verified that the treatments with the enzymes had not affected the integrity of the RNA (Fig 4A, lane 3 and 4). The products of such digestions were then employed to stimulate THP-1 cells in the presence of IFN-γ for 48 h. Then, the expression of MHC-I molecules was evaluated by flow cytometry. The preparations of DNase- and PK-digested RNA were still able to inhibit MHC-I expression in the same manner as intact RNA, indicating that contaminating DNA and proteins do not mediate the phenomenon of MHC-I inhibition (Fig 4B and 4C). *B*. *abortus* RNA was next digested with a prokaryotic RNA-specific RNase and this product was employed to stimulate THP-1 cells in the presence of IFN-γ for 48 h. The RNase used was RNase I from *Escherichia coli* which degrades single-stranded RNA in a mixture of mono-, di-, and tri-nucleotides. RNA preparations digested with RNase I completely lost the integrity of the RNA (Fig 4A, lane 5). Surprisingly, products from RNase I-digested RNA were still able to inhibit the IFN-γ-induced MHC-I surface expression to the same extent as intact RNA (Fig 4D and 4E). MHC-I down-modulation was not due to the presence of the RNase in the culture, since the negative control with merely RNase I was unable to reproduce the phenomenon. Overall, these results indicate that *B*. *abortus* RNA and its degradation products are the components involved in the inhibition of IFN-γ-induced MHC-I surface expression.

### *B*. *abortus* RNA down-modulates MHC-I in peripheral blood-isolated human monocytes and murine bone marrow-derived macrophages

**Fig 5:**
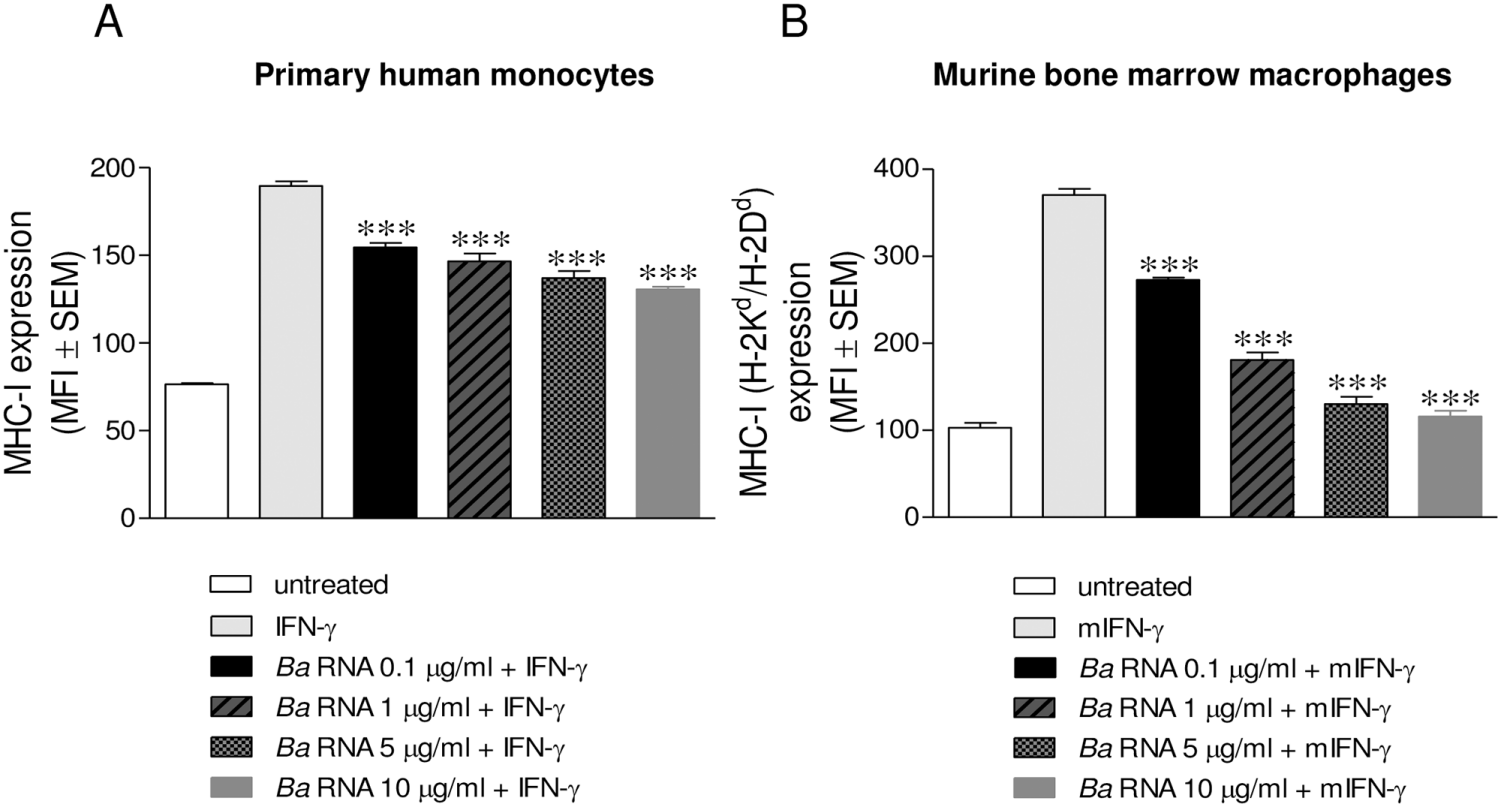
*B*. *abortus* RNA is able to down-modulate MHC-I on primary cultures of monocytes/macrophages. **(A and B)** Peripheral blood-isolated human monocytes (A) and murine bone marrow-derived macrophages (BMM) (B) were treated with different doses of *B*. *abortus* RNA. MHC-I expression was assessed by flow cytometry. Bars represent the arithmetic means ± SEM of five experiments. MFI, mean fluorescence intensity; mIFN-γ, murine IFN-γ. ***P<0.001 *vs*. IFN-γ-treated.

While THP-1 cells are a good model of human monocytes, we next evaluated whether our results could be extended to primary cultures of monocytes/macrophages. For this purpose,peripheral blood-isolated human monocytes or murine bone marrow-derived macrophages(BMM) were stimulated with different concentrations of *B*. *abortus* RNA and then the expression of MHC-I molecules was evaluated by flow cytometry. *B*. *abortus* RNA was significantly able to inhibit MHC-I expression in both human primary monocytes and murine BMM in a dose-dependent manner (Fig 5A and 5B). Thus, *B*. *abortus* RNA does not only inhibit MHC-I expression on THP-1 cells but also on human primary monocytes and murine macrophages.

### MHC-I down-modulation by *B*. *abortus* RNA on human monocytes is TLR8-mediated and involves the EGFR pathway

**Fig 6:**
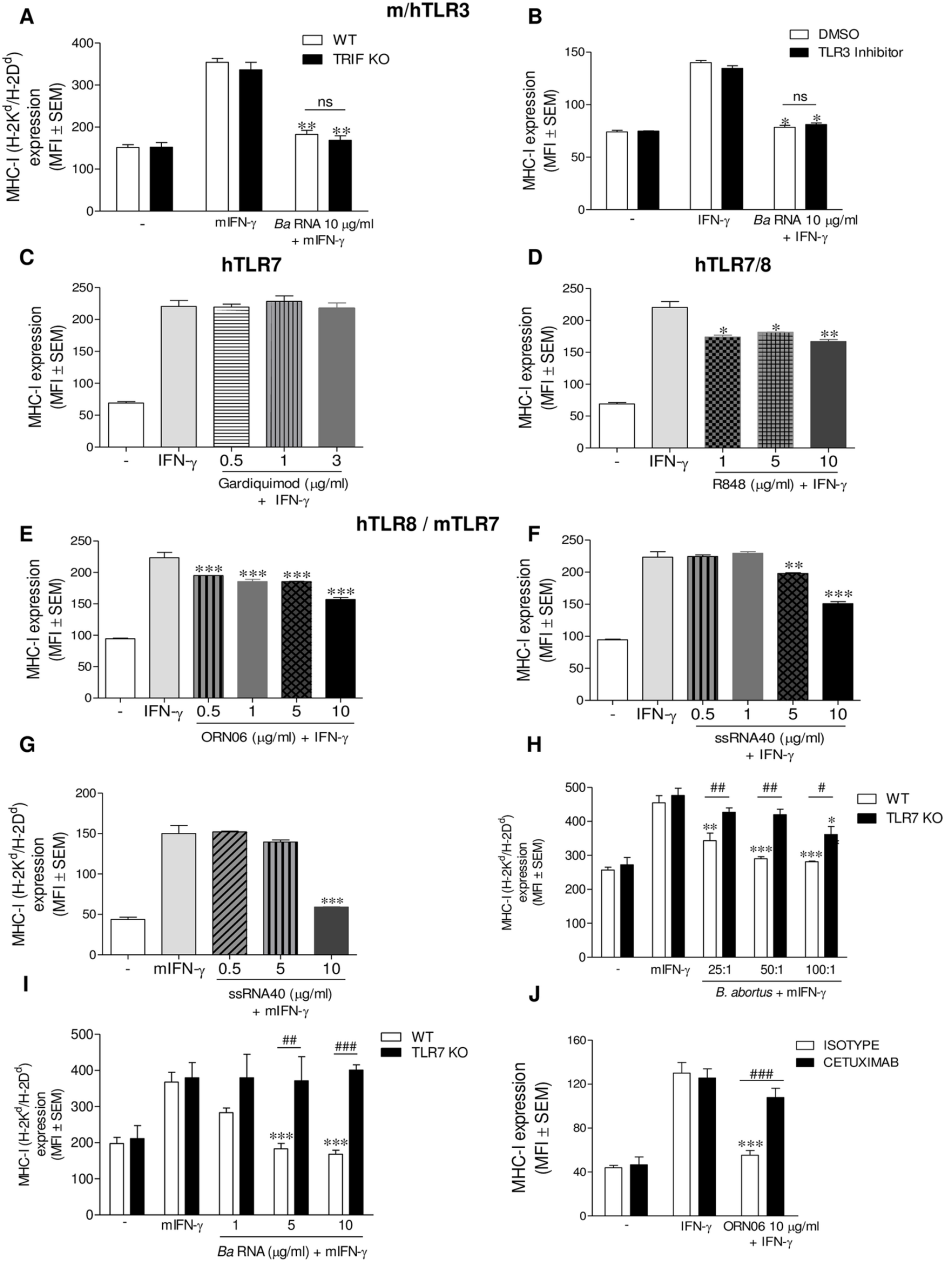
*B*. *abortus* RNA down-modulates MHC-I on human monocytes via TLR8 and through EGFR pathway. (A) Murine BMM purified from C57BL/6 WT or TRIF KO mice were stimulated with *B*. *abortus* RNA in the presence of IFN-γ for 48 h. (B) THP-1 cells were treated with *B*. *abortus* RNA 10 μg/ml in the presence of IFN-γ and hTLR3 inhibitor or vehicle (dimethyl sulfoxide (DMSO)) for 48 h. (C and D) THP-1 cells were treated with different doses of a hTLR7 agonist, Gardiquimod (C) or with different doses of a hTLR7 and hTLR8 agonist, R848 (Resiquimod) (D) in the presence of IFN-γ for 48 h. THP-1 cells (E and F) were treated with different doses of two hTLR8 agonists, ORN06 (E) and ssRNA40 (F) in the presence of IFN-γ for 48 h. (G) Murine BMM were treated with different doses of ssRNA40 in the presence of mIFN-γ for 48 h. (H and I) Murine BMM from TLR7 KO mice were infected with *B*. *abortus* (H) or stimulated with different doses of *B*. *abortus* RNA (I) in the presence of mIFN-γ for 48 h. (J) THP-1 cells were treated with ORN06 (10 μg/ml) in the presence of IFN-γ and Cetuximab or Isotype control for 48 h. MHC-I expression was assessed by flow cytometry. Bars represent the arithmetic means ± SEM of five experiments. MFI, mean fluorescence intensity; mIFN-γ, murine IFN-γ. *P<0.05; **P<0.01; ***P<0.001 *vs*. IFN-γ-treated. ^#^*P*<0.05; ^##^*P*<0.01; ^###^*P*<0.001 *vs*. WT or Isotype control, accordingly.

The most known receptors capable of detecting RNA are TLRs located in endosomes/phagolysosomes. Among them, TLR3 is capable of recognizing double-stranded RNA, TLR7 is capable of recognizing single-stranded RNA and TLR8 is also able to recognize single-stranded RNA and recently it was described as capable of recognizing RNA degradation products [13, 14]. Since the capacity of *Brucella* RNA to form secondary structures is still unknown and considering that TLR3 has been implicated in many functions mediated by viral double-stranded RNAs, we first wanted to evaluate whether TLR3 could be involved in *B*. *abortus* RNA-mediated inhibition of MHC-I molecules. TLR3 is the unique TLR that transduces its signal through the adapter protein TRIF. We therefore evaluated the effect of *B*. *abortus* RNA in BMM from TRIF KO mice. *B*. *abortus* RNA was able to inhibit the IFN-γ-induced MHC-I surface expression in BMM from TRIF KO mice to the same extent as in BMM from WT mice (Fig 6A). To confirm the fact that TLR3 was not involved in the inhibition mediated by *B*. *abortus* RNA we used a TLR3 inhibitor (TLR3/dsRNA Complex Inhibitor). Yet in the presence of a TLR3 inhibitor, *B*. *abortus* RNA down-regulated MHC-I expression confirming that TLR3 is not involved in this phenomenon (Fig 6B). Having discarded the participation of TLR3, we focused our attention on TLR7 and TLR8. Specific agonists have been described for either TLR7 or TLR8, or both. We used the human TLR7 (hTLR7) agonist Gardiquimod, the human TLR7/8 (hTLR7/8) agonist Resiquimod (R848) and the human TLR8 (hTLR8) agonists ssRNA40/LyoVec and ORN06/LyoVec. Gardiquimod was unable to inhibit the IFN-γ-induced MHC-I surface expression on THP-1 cells (Fig 6C). However, R848 was able to mimic the inhibition of MHC-I expression mediated by *B*. *abortus* RNA (Fig 6D). These results allowed us to discard hTLR7 and postulate hTLR8 as a possible receptor. To corroborate this, THP-1 cells were stimulated with the hTLR8 agonists ORN06 and ssRNA40. As shown in Fig 6E and 6F, both ORN06/LyoVec and ssRNA40/LyoVec were able to mimic the effect of *B*. *abortus* RNA on MHC-I surface expression. Although TLR8 is not functional in mice [16], it has been demonstrated that TLR7 performs its function [17, 18]. Since ssRNA40 is not only an agonist of hTLR8 but also of murine TLR7 (mTLR7) [17], we evaluated its effect on BMM. Corroborating our results, ssRNA40 was able to inhibit the expression of MHC-I in BMM (Fig 6G). To confirm these results BMM from WT or TLR7 KO mice were infected with *B*. *abortus* or stimulated with *B*. *abortus* RNA. Our results showed that the inhibition of MHC-I surface expression mediated by *B*. *abortus* and *B*. *abortus* RNA was abolished in BMM from TLR7 KO mice (Fig 6H and 6I). Altogether these results demonstrate that the MHC-I inhibition by *B*. *abortus* and its RNA is mediated by hTLR8/mTLR7.

Recently, we have demonstrated that the EGFR pathway is involved in the inhibition of MHC-I surface expression mediated by *B*. *abortus* infection [6]. In order to extend this finding and taking into account that TLR8 is the receptor involved in the phenomenon of *B*. *abortus* RNA-mediated MHC-I inhibition on human monocytes, we decided to evaluate the connection between TLR8 and EGFR signaling pathways. For this, THP-1 cells were stimulated with the hTLR8 agonist ORN06/LyoVec in the presence of an EGFR ligand-blocking antibody(Cetuximab). Neutralization of the EGFR significantly reversed the inhibition of MHC-I surface expression mediated by ORN06 (Fig 6J). This reversion was not due to a dysfunction of TLR8 caused by Cetuximab, as the secretion of pro-inflammatory cytokines downstream of NF-κB was unchanged between Isotype control and Cetuximab-treated cells (S7 Fig). Overall, our results indicate that *B*. *abortus* RNA inhibits the IFN-γ-induced MHC-I surface expression on human monocytes/macrophages by a TLR8-dependent mechanism and through the EGFR pathway.

### *B*. *abortus* RNA and its degradation products induce MHC-I intracellular retention within the Golgi apparatus

**Fig 7:**
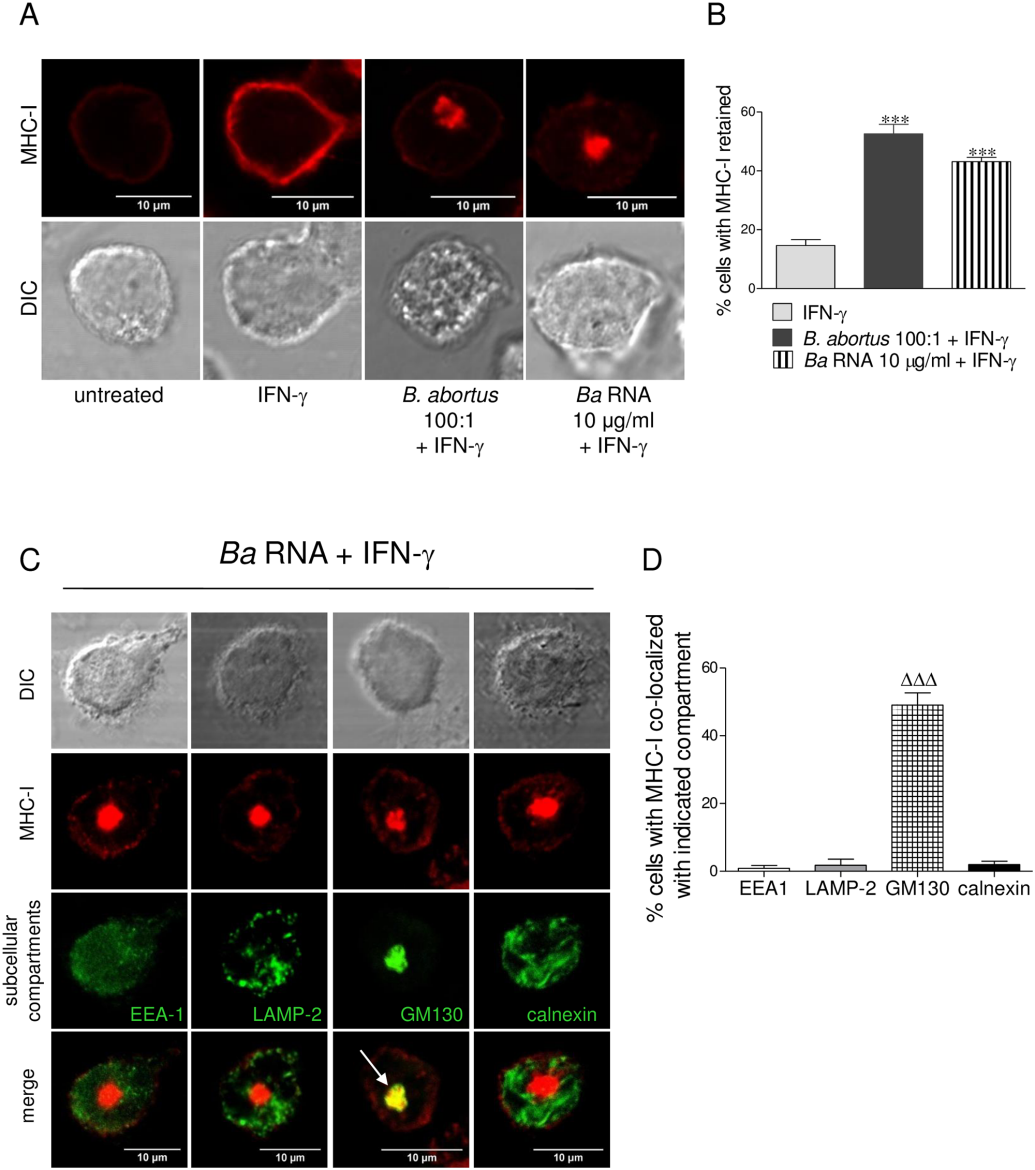
*B*. *abortus* RNA mimics MHC-I intracellular retention in Golgi apparatus mediated by *B*. *abortus* infection. (A) Confocal micrographs of THP-1 cells infected with *B*. *abortus* or treated with *B*. *abortus* RNA in the presence of IFN-γ for 48 h. MHC-I expression was determined with a primary anti-human MHC-I Ab (W6/32) and Alexa 546-labelled secondary Ab (red). (B) Quantification of MHC-I retention. Data are expressed as percentage of cells with MHC-I retained ± SEM of three independent experiments. The number of cells counted per experimental group was 200. (C) Confocal micrographs of THP-1 cells treated with *B*. *abortus* RNA in the presence of IFN-γ for 48 h. MHC-I expression was determined with a primary anti-human MHC-I Ab (W6/32) and Alexa 546-labelled secondary Ab (red). Subcellular localization markers were detected using mAbs specific for EEA1 (early endosomes), LAMP-2 (late endosomes/lysosomes), GM130 (Golgi) and calnexin (ER) followed by Alexa 488-labelled secondary Ab (green). White arrow shows co-localization (yellow staining). Results are representative of three independent experiments. (D) Quantification of co-localization of MHC-I with the subcellular compartments. Data are expressed as percentage of cells with MHC-I co-localized with indicated compartment ± SEM of three independent experiments. The number of cells counted per experimental group was 200. ***P<0.001 *vs*. IFN-γ-treated; ^ΔΔΔ^*P*<0.001 *vs*. the other subcellular compartments.

**Fig 8:**
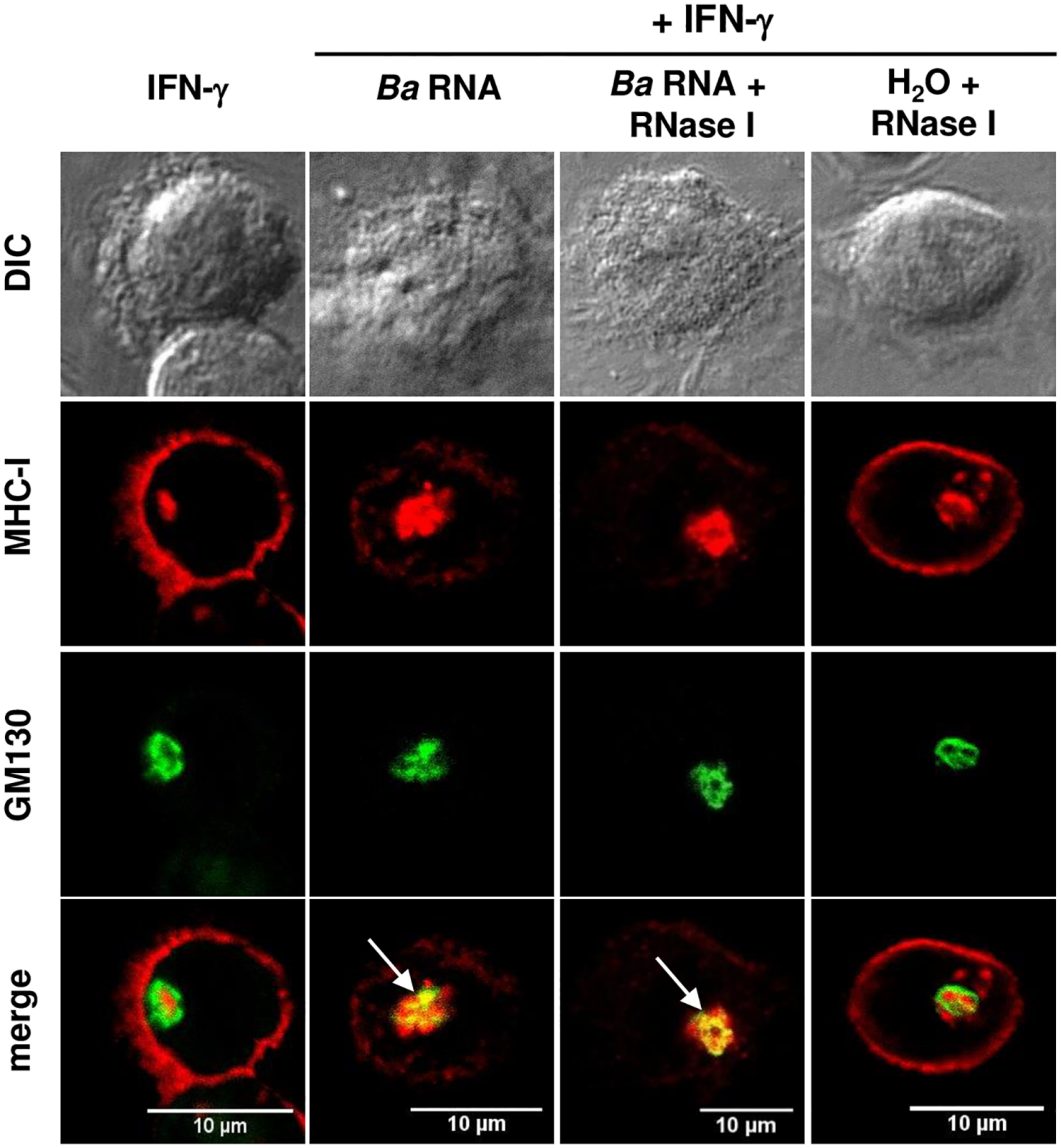
*B*. *abortus* RNA degradation products are also able to retain MHC-I within the Golgi apparatus. (A) Confocal micrographs of THP-1 cells treated with *B*. *abortus* RNA or RNase I-treated *B*. *abortus* RNA in the presence of IFN-γ for 48 h. MHC-I expression was determined with a primary anti-human MHC-I Ab (W6/32) and Alexa 546-labelled secondary Ab (red). Golgi apparatus was detected using a mAb specific for GM130 followed by Alexa 488-labelled secondary Ab (green). White arrows show co-localization (yellow staining). Cells treated only with RNase I were used as negative controls. Results are representative of three independent experiments.

We have previously demonstrated that *B*. *abortus* infection induces the intracellular retention of MHC-I molecules within the Golgi apparatus [5]. Thus, we evaluated whether *B*. *abortus* RNA was able to mimic this phenomenon. For this, the localization of MHC-I molecules was determined by confocal microscopy in cells infected with *B*. *abortus* or stimulated with *B*. *abortus* RNA in the presence of IFN-γ for 48 h. MHC-I expression was determined with an anti- HLA-ABC monoclonal antibody followed by Alexa 546-labelled secondary antibody. At 48 h of culture, cells treated only with IFN-γ showed MHC-I expression confined predominantly to the cellular membrane (Fig 7A). On the contrary, both *B*. *abortus*-infected monocytes as well as monocytes treated with *B*. *abortus* RNA, showed MHC-I expression restricted to the cellular interior concomitantly with a marked decrease of MHC-I surface expression (Fig 7A and 7B).Next, we examined the subcellular localization of retained MHC-I molecules. For this, THP-1 cells were treated with *B*. *abortus* RNA in the presence of IFN-γ for 48 h. MHC-I was detected as described previously and the subcellular compartments were detected with specific primary mAbs followed by Alexa 488-labelled secondary Ab. No colocalization was detected with either the early endosome marker EEA1, the lysosome marker LAMP-2 or the reticulum endoplasmic marker calnexin (Fig 7C). On the contrary, in 50% of the *B*. *abortus* RNA-treated monocytes that retained MHC-I, these molecules colocalized with the Golgi apparatus marker GM130 (Fig 7C and 7D). Taking into account that RNA degradation products were also able to inhibit the MHC-I surface expression, we next evaluated their capacity to induce intracellular retention of these molecules. Completely degraded RNA was able to induce the intracellular retention of MHC-I molecules within the Golgi apparatus to the same extent as intact RNA (Fig 8). Altogether these results demonstrate that *B*. *abortus* RNA and its degradation products mimic the intracellular retention of MHC-I within the Golgi apparatus observed with *B*. *abortus* infection.

### *B*. *abortus* RNA inhibits macrophages antigen presentation to CD8^+^ T lymphocytes via mTLR7

**Fig 9:**
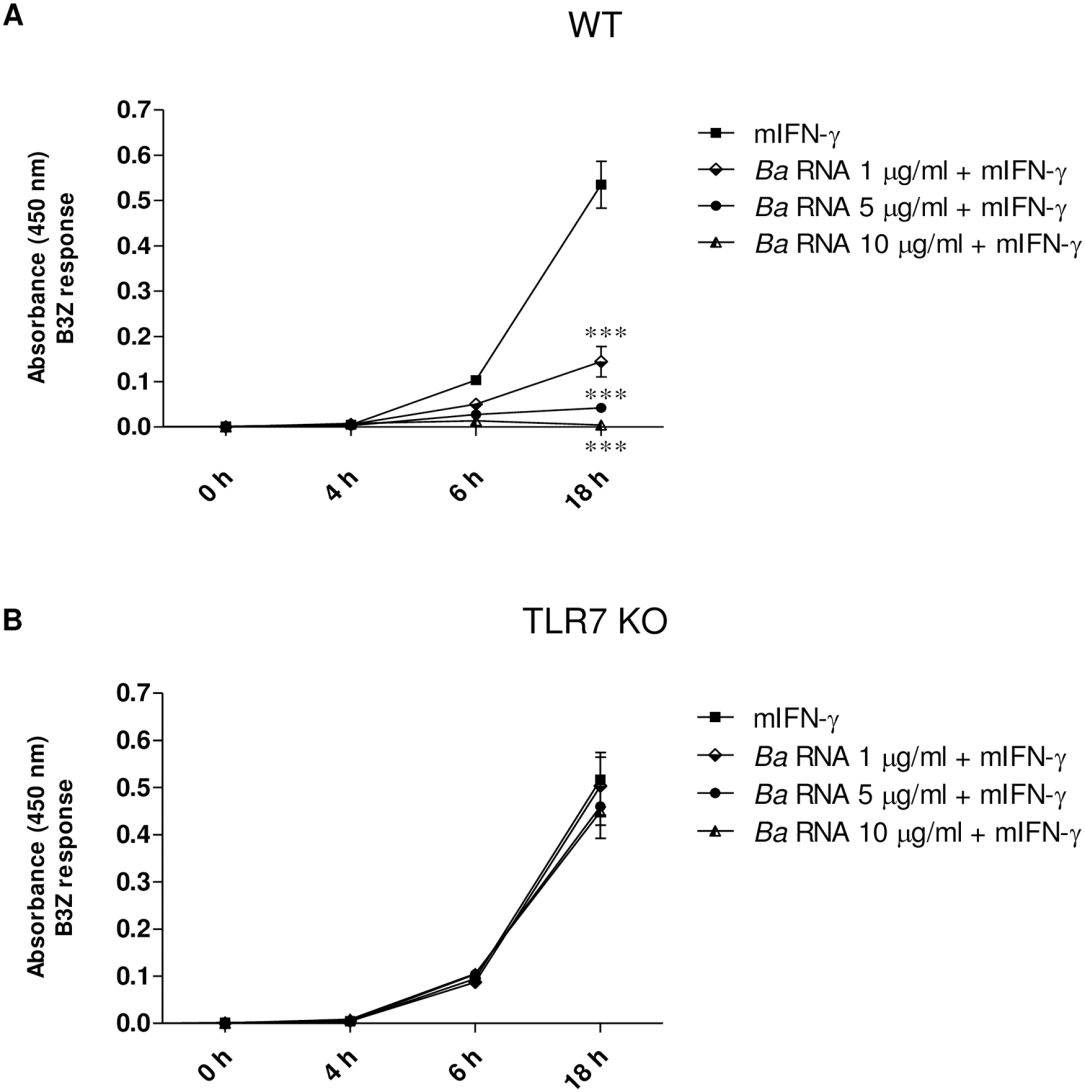
*B*. *abortus* RNA-mediated MHC-I inhibition correlates with diminished Ag presentation to MHC-I-restricted CD8^+^ T cells. BMM from WT (A) and TLR7 KO (B) mice were treated with different doses of *B*. *abortus* RNA in the presence of mIFN-γ for 48 h. Then cells were washed and incubated with 20 ng/ml of 257–264 OVA peptide (SIINFEKL) for 20 min at 37°C. BMM from WT (A) and TLR7 KO (B) mice were washed and cultured for 0, 4, 6 and 18 h at 37°C with B3Z cells, a T cell hybridoma specific for OVA-K^b^, which carries a β-galactosidase construct driven by NF-AT elements from the IL-2 promoter. T cell activation was measured using a colorimetric assay for LacZ activity with *o*-nitrophenyl-P-D-galactoside as a substrate. Background absorbance values obtained for BMM cultured in the absence of OVA were subtracted. ***P<0.001 *vs*. mIFN-γ-treated.

Finally, we evaluated whether the diminished MHC-I surface expression induced by *B*. *abortus* RNA was associated with changes in Ag presentation to MHC-I-restricted CD8^+^ cytotoxic T cells. For this, murine BMM from WT and TLR7 KO mice were treated with different doses of *B*. *abortus* RNA in the presence of murine IFN-γ (mIFN-γ) for 48 h, followed by incubation with OVA peptide (SIINFEKL) and a B3Z T-cell hybridoma specific for OVA-K^b^, which carries a β-galactosidase construct driven by NF-AT elements from the IL-2 promoter. BMM from WT and TLR7 KO mice treated solely with mIFN-γ presented K^b^-restricted OVA peptide efficiently after 6 h and onwards (Fig 9A and 9B), as evidenced by the ability of these cells to induce LacZ activity in B3Z cells. Treatment of BMM from WT mice with *B*. *abortus* RNA (1–10 μg/ml) in the presence of mIFN-γ significantly inhibited presentation of OVA peptide since it diminished the response of B3Z cells, compared to mIFN-γ-only treated cells (Fig 9A). However, treatment of BMM from TLR7 KO mice with *B*. *abortus* RNA did not affect antigen presentation to CD8^+^ T lymphocytes compared to mIFN-γ-only treated cells (Fig 9B). Taken together, these results indicate that inhibition of MHC-I expression by *B*. *abortus* RNA correlates with diminished Ag presentation to MHC-I-restricted CD8^+^ cytotoxic T cells. In addition, our results demonstrate that inhibition of Ag presentation to CD8^+^ T cells by *B*. *abortus* RNA is mediated by mTLR7.

## Methods

### Ethics statement

Human monocytes were isolated exclusively from healthy adult blood donors in accordance with the guidelines of the Ethical Committee of the IMEX Institute. All adult blood donors provided their informed written consent prior to the study. Mouse bone marrow-derived macrophages (BMM) were generated by differentiation of bone marrow progenitors from female C57BL/6 mice (aged 2–3 months). Mice were kept under specific pathogen-free conditions in positive-pressure cabinets and provided with sterile food and water ad libitum. All animal procedures were performed according to the rules and standards for the use of laboratory animals of the National Institutes of Health. Animal experiments were approved by the Animal Care and Use Committee of the IMEX Institute. The protocol license number assigned by this Committee is: 020/2016.

### Bacteria

*B*. *abortus* S2308, *Salmonella typhimurium* SL14028, *Bacillus cereus* B10502, *Escherichia coli* 11105 and *Klebsiella pneumoniae* 700603, and *B*. *abortus* RB51, *B*. *abortus virB10*, *B*. *abortus btpA*, *B*. *abortus btpB*, *B*. *abortus btpAbtpB* or *B*. *abortus Bpe159* mutant strains were cultured in tryptose-soy agar supplemented with yeast extract (Merck). The number of bacteria on stationary-phase cultures was determined by comparing the OD at 600 nm with a standard curve. To obtain heat-killed *B*. *abortus* strains, bacteria were washed in PBS, heat killed at 70°C for 20 min and stored at -70°C until used. Total absence of *B*. *abortus* viability subsequent to heat killing was verified by the absence of bacterial growth in tryptose-soy agar. All live *Brucella* manipulations were performed in biosafety level 3 facilities, located at the Instituto de Investigaciones Biomédicas en Retrovirus y SIDA (Buenos Aires, Argentina).

### Expression and purification of recombinant lipidated Omp19 (L-Omp19) and unlipidated Omp19 (U-Omp19) from *B*. *abortus*

Lipoproteins were expressed and purified as previously described [19]. To eliminate LPS contamination, recombinant Omps were adsorbed with Sepharose-polymyxin B (Sigma-Aldrich). Both proteins contained less than 0.25 endotoxin U/μg of protein as assessed by Limulus Amebocyte Lysate assay (Lonza). The protein concentration was determined by the BCA protein assay (Pierce) using bovine serum albumin as standard. The purified proteins were aliquoted and stored at -70°C until used.

### LPS and DNA from *B*. *abortus*

*B*. *abortus* 2308 LPS was provided by I. Moriyón (University of Navarra, Pamplona, Spain). The purity and characteristics of these preparations have been described elsewhere [20]. LPS was solubilized in water by sonication at the appropriate concentration and autoclaved before use. *B*. *abortus* DNA was purified by extraction with phenol:chloroform:isoamyl alcohol and ethanol precipitation [21]. To eliminate LPS contamination, DNA was adsorbed with Sepharose-polymyxin B (Sigma-Aldrich). DNA contained less than 0.25 endotoxin U/μg of protein as assessed by Limulus Amebocyte Lysate assay (Lonza).

### Cells and media

All experiments were performed at 37°C in 5% CO_2_ atmosphere and standard medium composed of RPMI-1640 supplemented with 25 mM Hepes, 2 mM L-glutamine, 10% heat-inactivated fetal bovine serum (Gibco), 100 U of penicillin/ml and 100 μg of streptomycin/ml. THP-1 cells were obtained from the American Type Culture Collection (Manassas, VA) and cultured as previously described [19]. To induce maturation, cells were cultured in 0.05 μM 1,25-dihydroxyvitamin D_3_ (EMD Millipore) for 72 h. Peripheral blood mononuclear cells (PBMCs) were obtained by Ficoll-Hypaque (GE Healthcare) gradient centrifugation from human blood collected from healthy adult individuals. Monocytes were then purified from PBMCs by Percoll (GE Healthcare) gradient and resuspended in standard medium. Purity of the isolated CD14^+^ monocytes was more than 80% as determined by flow cytometry. Viability of cells was more than 95% in all the experiments as measured by trypan blue exclusion test. Mouse bone marrow-derived macrophages (BMM) were generated by differentiation of bone marrow progenitors from C57BL/6 wild type mice, TRIF KO or TLR7 KO mice (provided by Federal University of Minas Gerais, Belo Horizonte, Brazil) with rM-CSF (PeproTech), as previously described [22].

### *In vitro* infection

THP-1 cells at a concentration of 0.5 x 10^6^/ml were infected in round-bottom polypropylene tubes (Falcon) with different multiplicities of infection (MOI) of *B*. *abortus* S2308, *B*. *abortus* RB51, *B*. *abortus virB10*, *B*. *abortus btpA*, *B*. *abortus btpB*, *B*. *abortus btpAbtpB* or *B*. *abortus Bpe159* mutants. All infections were done in the presence of 150 U/ml IFN-γ (Endogen) for 2 h in standard medium containing no antibiotics. In another set of experiments, BMM from WT or TLR7 KO mice at a concentration of 0.5 x 10^6^/ml were infected in a 24-well plate with different MOI of *B*. *abortus* S2308. Infections were done in the presence of 10 ng/ml mIFN-γ (Peprotech) for 2 h in standard medium containing no antibiotics. In all cases, cells were extensively washed to remove uninternalized bacteria and infected cells were maintained in culture in the presence of IFN-γ or mIFN-γ, 100 μg/ml gentamicin and 50 μg/ml streptomycin for an additional 48 h.

### Apoptosis assay

For viability assay, THP-1 cells at a concentration of 0.5 x 10^6^/ml were treated with different doses of *B*. *abortus* RNA in the presence of IFN-γ for 48 h. THP-1 cells treated with 2% paraformaldehyde (PFA) were also included as positive control. After 48 h, cells were washed and incubated with Annexin V-FITC and Propidium Iodide (BD Biosciences) for 10 min on ice in darkness. Then, cells were evaluated in the quadrants of Annexin V^+^/PI^-^ (early apoptosis), Annexin V^+^/PI^+^ (late apoptosis) and Annexin V^-^/PI^+^ (necrosis). After labelling, cells were analyzed on a FACSCalibur flow cytometer (BD Biosciences) and data were processed using CellQuest software (BD Biosciences).

### RNA preparation

5–10 x 10^6^ PBMCs or 5 x 10^8^ CFU were resuspended in 1ml of TRIzol Reagent (Invitrogen) and total RNA was extracted according to the manufacturer’s instructions. OD at 260 was measured for RNA quantification. In another set of experiments, *B*. *abortus* RNA was purified with *Quick*-RNA MiniPrep (Zymo Research) according to the manufacturer’s instructions. The purity of *B*. *abortus* RNA was assessed using a DeNovix DS-11 Spectrophotometer (DeNovix Inc.) with a ratio of absorbance 260/280 > 2.0 and a ratio of absorbance 260/230 > 1.8. In one set of experiments, RNA was treated with DNase RQ1 (Promega), Proteinase K (PK) (Promega) or *E*. *coli* RNase I (Life Technologies) prior to cell stimulation. RNA preparations (*B*. *abortus* RNA, DNase-treated *B*. *abortus* RNA, PK-treated *B*. *abortus* RNA and RNase I-treated *B*. *abortus* RNA) were further visualized by 1% agarose gel electrophoresis. The RNA was detected using UV light and the size of the RNA was determined using standard 100 bp Plus DNA ladder (TransGen Biotech Co., Ltd.).

### *In vitro* stimulation

Cells at 0.5 x 10^6^/ml were stimulated with *B*. *abortus* RNA, other prokaryotic or eukaryotic RNAs, DNase-treated *B*. *abortus* RNA, PK-treated *B*. *abortus* RNA, RNase I-treated *B*. *abortus* RNA, HK *B*. *abortus* strains, different structural components of *B*. *abortus* (LPS, DNA and lipoproteins) or TLR ligands in the presence of 150 U/ml IFN-γ for 48 h in standard medium containing antibiotics. In another set of experiments, THP-1 cells were treated with *B*. *abortus* RNA complexed with Lipofectamine 2000 (Invitrogen). Briefly, Lipofectamine was mixed with bacterial RNA (1:3 ratio) in 100 μL/well serum-free RPMI and incubated for 20 min at room temperature. Then, complexes were added to the cells in the presence of 1.25% FBS and cell cultures were incubated for 48 h at 37°C in a 5% CO_2_ atmosphere. In all cases, MHC-I expression was evaluated by flow cytometry.

### Flow cytometry

After *B*. *abortus* infection or stimulation; THP-1 cells or human primary monocytes were stained with FITC-labelled anti-human HLA-ABC (clone G46-2.6; BD Pharmingen) or isotype-matched control mAbs. In the experiments with murine macrophages, BMM were infected with *B*. *abortus*, or treated with *B*. *abortus* RNA or TLR ligands in the presence of 10 ng/ml recombinant murine IFN-γ (PeproTech) for 48 h. To determine MHC-I surface expression, cells were stained with PE- or FITC-labelled anti-mouse H-2K^d^/H-2D^d^ (clone 34-1-2S; BioLegend). In all cases, cells were washed and incubated with 7-Amino-Actimycin D (7-AAD; BD Biosciences) for 10 min on ice in darkness. MHC-I expression was evaluated gating on viable cells (7-AAD negative cells). After labelling, cells were analyzed on a FACSCalibur flow cytometer (BD Biosciences) and data were processed using CellQuest software (BD Bioscience) or FlowJo 7.6 software.

### Confocal microscopy

THP-1 cells were incubated in chambers-slides (2 x 10^5^ cells/well) with 10 ng/ml PMA (Sigma-Aldrich) for 24 h to promote adherence. Then, cells were infected with *B*. *abortus* or stimulated with *B*. *abortus* RNA or RNase I-treated *B*. *abortus* RNA in the presence of IFN-γ for 48 h, fixed with 2% paraformaldehyde, permeabilized with 0.1% saponin and incubated with anti-HLA-ABC class I mAb W6/32, (purified from murine hybridoma culture supernatants) and Alexa 546-labelled secondary Ab (Invitrogen). Subcellular compartments were detected using mAbs specific for EEA1 (early endosomes), LAMP-2 (late endosomes/lysosomes), GM130 (Golgi) and calnexin (ER) (BD Biosciences) following Alexa 488-labelled secondary Ab (Invitrogen). Slides were mounted with PolyMount (Polysciences) and analyzed using FV-1000 confocal microscope with an oil-immersion Plan Apochromatic 60X NA1.42 objective (Olympus).

### Ag cross-presentation assay

Presentation of OVA epitope 257–264 on K^b^ (SIINFEKL) was detected using the T cell hybridoma B3Z, which carries a β-galactosidase construct driven by NF-AT elements from the IL-2 promoter [23]. For Ag presentation assays, *B*. *abortus* RNA-treated BMM from WT or TLR7 KO mice were exposed to 20 ng/ml of the SIINFEKL epitope during 20 min at 37°C. Then cells were washed, suspended in complete medium, and cultured in the presence of the T cell hybridoma B3Z. After 0, 4, 6 and 18 h of culture, cells were washed with PBS, and the cross-presentation was evaluated by a colorimetric assay using *o*-nitrophenyl-p-D-galactoside (ONPG) (Sigma-Aldrich) as substrate to detect the LacZ activity in B3Z lysates.

### Reagents

Antibody targeting EGFR (Cetuximab) was purchased from Merck Serono. Gardiquimod, R848 (Resiquimod), ssRNA40/LyoVec and ORN06/LyoVec were purchased from InvivoGen. The 257–264 OVA peptide (SIINFEKL) was provided by Dr. S. Amigorena (Institut Curie, Paris, France). TLR3/dsRNA Complex Inhibitor was purchased from Calbiochem.

### Measurement of cytokine concentrations

Human TNF-α and IL-1β were measured in culture supernatants by sandwich ELISA, using paired cytokine-specific mAbs according to the manufacturer’s instructions (BD Pharmingen).

### Statistical analysis

Results were analyzed with one-way ANOVA followed by *post hoc* Tukey test using the GraphPad Prism software.

## Discussion

*B*. *abortus* is an intracellular pathogen capable of surviving inside macrophages [24]. The success of *B*. *abortus* as a chronic pathogen relies on its ability to orchestrate different strategies to evade the adaptive CD8^+^ T cells responses that it elicits. Previously, we have demonstrated that *B*. *abortus* infection inhibits the IFN-γ-induced MHC-I surface expression on human monocytes down-modulating cytotoxic CD8^+^ T cell responses [5]. Moreover, we have recently deepened into various aspects of this event, such as its kinetics and the participation of the EGFR pathway [6]. Two striking features of the phenomenon of MHC-I inhibition allowed us to shed light on the *B*. *abortus* components involved. First of all, heat-killed *B*. *abortus* is incapable of inhibiting MHC-I expression [5]. Secondly, the phenomenon is triggered early during infection [6]. Together, these results indicated that only metabolically active viable bacteria can inhibit MHC-I expression and that it must occur during the time span before the bacteria are removed and/or mediated by a product generated early in response to infection.

In this study, we could corroborate that what *Brucella* employs to inhibit MHC-I on monocytes/macrophages is a component associated with bacterial viability itself regardless of the most relevant bacterial virulence factors. Particularly, we elucidated that this component is *B*. *abortus* RNA. Moreover, our experiments demonstrated that not only wild-type *B*. *abortus* RNA but also the RNA of two mutants strains, RB51 and *virB10*, were equally able to inhibit MHC-I. These results together with those shown in Fig 1 and S1 Fig corroborate that what the cells sense is a general determinant of bacterial viability different from its virulence factors.Supporting these results, we demonstrated that the inability of heat-killed *B*. *abortus* to inhibit MHC-I surface expression is due to the absence of RNA in these bacterial preparations. In line with this evidence, it is widely known that live vaccines trigger more vigorous immune responses than their killed counterparts, even when live microorganisms are attenuated by elimination of their virulence factors [25]. Since structural bacterial components are present in both live and dead microorganisms, this suggested that there should be non-characterized determinants in live bacteria important for the induction of an effective protective immune response. In this sense, it has been demonstrated that macrophages can directly sense microbial viability through detection of prokaryotic messenger RNA (mRNA), a *vita*-PAMP present only in viable bacteria, triggering a unique innate and a robust adaptive antibody responses[10]. Notably, the innate response evoked by viability and prokaryotic mRNA was thus far considered to be reserved for pathogenic bacteria, but Sander *et al* in their study show that even non-pathogenic bacteria in sterile tissues can trigger similar responses, provided they are alive [10]. Furthermore, our results also demonstrated that inhibition of MHC-I is not restricted to *B*. *abortus* as it could be extended to other prokaryotic RNAs, suggesting the broad implications of this immune regulation in the context of other infectious processes.

One issue that merits discussion is how, during *B*. *abortus* infection, the human monocyte/macrophages are able to sense bacterial RNA. This question can be answered if we review the biology of *B*. *abortus* and the localization of receptors capable of detecting RNA. It has been described that viral and bacterial RNA are sensed by pattern recognition receptors (PRRs), among which the TLRs family has gained more attention [26, 27]. TLR3, TLR7 and TLR8 are the ones preferentially expressed in intracellular vesicles of the endoplasmic reticulum (ER), endosomes, and lysosomes [28]. With respect to the intracellular cycle of the bacterium, *B*. *abortus* is able to enter, survive and replicate within vacuolar phagocytic compartments of macrophages [29]. Once inside the macrophages, *Brucella* dwells in an acidified compartment that fuses with components of the early and late endosomal/lysosomes pathway [7, 30]. There, the vast majority of the ingested bacteria are rapidly killed. However, the establishment of a persistent infection depends on the ability of the bacterium to form a *Brucella*-containing vacuole (BCV), which traffics from the endocytic compartment to the endoplasmic reticulum (ER) [7, 29, 31]. Once inside the replicative BCV, bacteria are resistant to further attack and begin to multiply dramatically [7, 31]. Starr *et al* demonstrated that *Brucella* replication in the ER is followed by BCV conversion into multi-membrane LAMP-1-positive vacuoles with autophagic features (aBCV). Furthermore, aBCVs were required to complete the intracellular *Brucella* lifecycle and for cell-to-cell spreading [32]. In this context, it is possible that while *B*. *abortus* traffics through early and late endosomes/lysosomes the bacterial RNA released during phagocytosis activate endolysosomal TLRs. On the other hand, *B*. *abortus* mutant strains on virulence factors are also capable of infecting human monocytes/macrophages and transiting by early and late endosomes/lysosomes, but, unlike wild-type *B*. *abortus*, they are unable to replicate in BCVs and thus persist into the cell host. However, our results demonstrated that these strains are equally able to down-regulate MHC-I than wild-type *B*. *abortu*s. As a consequence, the RNA of these bacteria could also gain access to TLR3, TLR7 or TLR8 in their transit through endosomes and lysosomes, although they do not persist in macrophages. In accordance with this, it was reported that human TLR8 is activated upon recognition of *Borrelia burgdorferi* RNA in the phagosome of human monocytes [33]. Furthermore, in line with endosomal TLRs sensing in an infectious context, we have previously demonstrated that MHC-I inhibition is an early event during infection, already observed at 8 h post-infection [6]. This goes along with the time that elapses in the passage of the bacteria by the early and late endocytic/lysosomal pathway [30]. In our *in vitro* experiments of stimulation with purified RNA, either in the presence or the absence of transfection, the entry of RNA by endocytosis gaining access to the endosomal TLRs can perfectly mimic what happens in an infectious context.

Regarding the receptor involved, our results indicated that an hTLR7/8 agonist such as R848 was able to mimic the MHC-I inhibition mediated by *B*. *abortus* RNA. However, an hTLR7 agonist *per se* was unable to reproduce MHC-I inhibition. This led us to propose hTLR8 as a possible receptor, which was corroborated by the specific human TLR8 agonists ssRNA40 and ORN06. The greater efficiency of the synthetic oligonucleotide ORN06 in MHC-I inhibition may be due to the fact that it contains 6 repeats of the UUGU sequence motif, identified as the minimal motif responsible for ssRNA40 immunoactivity [34]. In addition, the involvement of hTLR8 in the *B*. *abortus* RNA-mediated MHC-I inhibition was corroborated in murine BMM using the agonist ssRNA40 which is also specific for murine TLR7, since TLR7 acts as the human TLR8 in mice [17, 18]. Moreover, our results with BMM from TLR7 KO mice confirm that the inhibition of MHC-I surface expression by *B*. *abortus* and its RNA is mediated by hTLR8/mTLR7. In agreement with these results, the inhibition of antigen presentation to CD8^+^ T cells by *B*. *abortus* RNA was abolished in BMM from TLR7 KO mice. Further experiments *in vivo* are needed to determine the involvement of TLR8/TLR7 in the cytotoxic CD8^+^ T cell responses and chronicity of *B*. *abortus*-infected mice.

Single-stranded RNA has been identified as the natural ligand of TLR7 and TLR8 [17, 35]. Of note, a recent report identifying the molecular structure of TLR8 showed that this receptor recognizes degradation products of RNA, specifically an uridine mononucleoside at one binding site and oligonucleotides like UG or UUG at a distinct second binding site [14]. The concept of recognition of RNA degradation products by TLR8 raises the hypothesis that phosphatases and/or nucleases of bacterial or host origin might play a role upstream of TLR8 activation [36], in analogy to the requirement for lysosomal endonuclease DNase II for the activation of TLR9 [37, 38]. In agreement with this evidence, we could observe that RNA digested by a specific prokaryotic RNase was able to inhibit MHC-I in the same manner as intact RNA. These results indicate that these degradation products could be sensed by TLR8.

Although our results indicated that human TLR3 and TLR7 are not involved in MHC-I inhibition, Campos *et al* have recently demonstrated that both receptors play an important role in sensing *B*. *abortus* RNA to induce the production of pro-inflammatory cytokines and type I IFN expression in murine DCs. However, these receptors were not required to control *Brucella* infection *in vivo* [39]. To explain the latter, they hypothesized that TLR13, a PRR involved in sensing a specific sequence from bacterial 23S rRNA [40, 41], could play a role in *B*. *abortus* RNA sensing. However, we did not focus our attention on TLR13 since it is a receptor present in mice but not in humans [40, 41]. Beyond TLRs, RIG-I and MDA5 have been characterized as cytosolic receptors capable of sensing RNA. More specifically, MDA5 was identified to initiate antiviral signaling in response to long stretches of viral double-stranded RNA, whereas RIG-I is a sensor of short double-stranded or single-stranded RNA with 5’-triphosphate termini [42, 43]. Moreover, RIG-I was also involved in the recognition of bacterial RNA. It has been demonstrated that RIG-I detects infection with live *Listeria monocytogenes* by sensing the RNA secreted into the cytosol of infected cells [44, 45]. Although RIG-I and MDA5 could also be involved in the MHC-I inhibition mediated by *B*. *abortus* RNA, taking into account the cytosolic location of such receptors, the RNA should be able to be transferred from the phagosomes into the cell cytosol. However, this does not occur for all bacteria as it was demonstrated for *B*. *burgdorferi* RNA [33]. Regarding *Brucella*, it was demonstrated that *B*. *abortus* DNA can activate cytosolic molecules such as AIM2 and STING [46, 47]; however, whether RNA might gain access to cytosolic receptors during phagocytosis has not been investigated yet.

We have previously demonstrated that the EGFR pathway is involved in the inhibition of MHC-I mediated by *B*. *abortus* infection [6]. Moreover, that EGF and TGF-α are EGF-like ligands involved in the phenomenon of MHC-I inhibition [6]. In light of the results that we obtained in this study and those recently published [6], we next investigated if there was a connection between RNA sensed by TLR8 and the EGFR pathway. EGFR neutralization with Cetuximab led to a partial reversion of the TLR8 agonist mediated-MHC-I inhibition suggesting a clear connection between the TLR8 and EGFR pathways. To our knowledge, this is the first report describing a link between these pathways.

**Fig 10:**
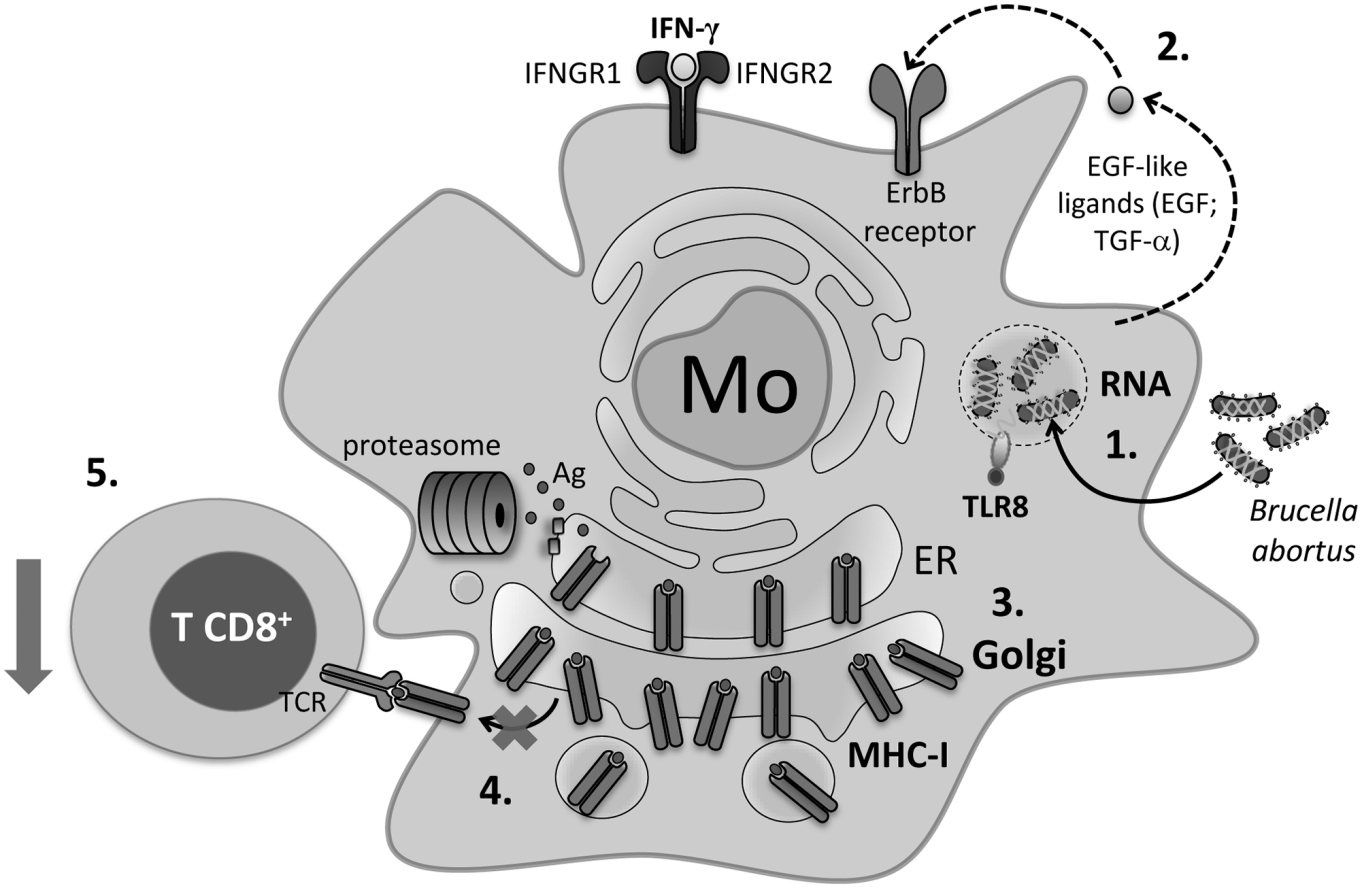
Proposed model for the MHC-I surface down-regulation mechanism mediated by *B*. *abortus*. 1. Infection of human monocytes/macrophages with *B*. *abortus* induces the release of its RNA and RNA degradation products into the *Brucella*-containing endosomes. 2. These molecules via TLR8 induce the secretion of EGF-like ligands such as EGF and TGF-α which bind ErbB receptors on the cell surface causing their activation. 3. These effects finally cause the retention of MHC-I molecules within the Golgi apparatus. 4. MHC-I molecules are therefore unable to reach the cell surface and present bacterial Ags to CD8^+^ T cells. 5. Inhibition of Ag presentation enables the bacteria to hide inside human monocytes/macrophages and avoid the cytotoxic CD8^+^ T cell responses.

Overall, the results obtained in this study support a model in which infection with *B*. *abortus* induces the release of its RNA and RNA degradation products into the *Brucella*-containing endosomes. These molecules via TLR8 induce the secretion of EGF-like ligands such as EGF and TGF-α which bind ErbB receptors on the cell surface causing their activation. These effects finally lead to the retention of MHC-I molecules within the Golgi apparatus. MHC-I molecules are therefore unable to reach the cell surface and present bacterial Ags to CD8^+^ T cells (Fig 10).

Here we elucidate that the *vita*-PAMP RNA is a component employed by *B*. *abortus* to inhibit MHC-I expression, an event whereby the bacteria could avoid the cytotoxic CD8^+^ T cell immunological surveillance establishing a chronic infection.

## Supporting information

S1 Fig Only viable *B*. *abortus*, independently of its virulence factors, is able to inhibit MHC-I expression. (A and B, Panels i and iii) THP-1 cells were infected with *B*. *abortus* RB51 (A) or *virB10*^-^ (B) at different MOI in the presence of IFN-γ for 2 h, washed and cultured in the presence of IFN-γ for 48 h. (A and B, Panels ii and iv) At the same time, heat-killed bacteria (HK) were used to treat THP-1 cells in the presence of IFN-γ for 48 h. MHC-I expression was assessed by flow cytometry. Bars represent the arithmetic means ± SEM of five experiments. MFI, mean fluorescence intensity. ***P<0.001 *vs*. IFN-γ-treated.
(TIF)

S2 Fig MiniPrep-purified *B*. *abortus* RNA is also able to down-modulate MHC-I. THP-1 cells were treated with different doses of *B*. *abortus* RNA purified with *Quick*-RNA MiniPrep kit in the presence of IFN-γ for 48 h. MHC-I expression was assessed by flow cytometry. Bars indicate the arithmetic means ± SEM of five independent experiments. MFI, mean fluorescence intensity. **P<0.01 *vs*. IFN-γ-treated.
(TIF)

S3 Fig RNA is absent in heat-killed *B*. *abortus*. (A) RNA was purified from *B*. *abortus* or HKBA. In addition, *B*. *abortus* RNA was heat-treated at 70°C for 20 min. Each preparation was visualized by 1% agarose gel electrophoresis. (B) THP-1 cells were stimulated with *B*. *abortus* RNA, RNA extraction products from HKBA or heat-treated *B*. *abortus* RNA in the presence of IFN-γ for 48 h. MHC-I expression was assessed by flow cytometry. Bars represent the arithmetic means ± SEM of three experiments. MFI, mean fluorescence intensity. **P<0.01 *vs*. IFN-γ-treated.
(TIF)

S4 Fig *B*. *abortus* RNA complements its absence in HKBA, making it capable of down-modulating MHC-I. THP-1 cells were treated with HKBA with or without *B*. *abortus* RNA in the presence of IFN-γ for 48 h. MHC-I expression was assessed by flow cytometry. Bars indicate the arithmetic means ± SEM of five independent experiments. MFI, mean fluorescence intensity. **P<0.01 *vs*. IFN-γ-treated.
(TIF)

S5 Fig Nystatin reversed MHC-I inhibition mediated by *B*. *abortus* RNA. THP-1 cells were treated with *B*. *abortus* RNA (10 μg/ml) in the presence of IFN-γ and in the presence or absence of an endocytosis inhibitor (Nystatin) for 48 h. MHC-I expression was assessed by flow cytometry. Bars indicate the arithmetic means ± SEM of five independent experiments. MFI, mean fluorescence intensity. *P<0.05 *vs*. IFN-γ-treated; ^##^ *P*<0.01 *vs*. Control.
(TIF)

S6 Fig Other prokaryotic but not eukaryotic RNAs are also able to inhibit IFN-γ-induced MHC-I expression. (A-D) THP-1 cells were treated with different doses of *S*. *typhimurium* (A), *B*. *cereus* (B), *E*. *coli* (C) or *K*. *pneumoniae* (D) RNAs in the presence of IFN-γ for 48 h. (E) THP-1 cells were treated with different doses of PBMCs RNA in the presence of IFN-γ for 48 h. MHC-I expression was assessed by flow cytometry. Bars indicate the arithmetic means ± SEM of three independent experiments. MFI, mean fluorescence intensity. *P<0.05; **P<0.01; ***P<0.001 *vs*. IFN-γ-treated.
(TIF)

S7 Fig Cetuximab does not affect the functionality of TLR8. (A and B) THP-1 cells were treated with ORN06 or *B*. *abortus* RNA in the presence of Cetuximab or Isotype control for 24 h. Supernatants were then collected and the amount of TNF-α (A) or IL-1β (B) was determined by ELISA. Bars indicate the arithmetic means ± SEM of three independent experiments. ***P<0.001 *vs*. untreated + Isotype or Cetuximab, accordingly.
(TIF)
