# Supplementary figures and images for: B. abortus RNA is the component involved in the down-modulation of MHC-I expression on human monocytes via TLR8 and the EGFR pathway
Source: PLoS Pathog. 2017 Aug 2;13(8):e1006527. doi: 10.1371/journal.ppat.1006527 (PMC5540288; doi:10.1371/journal.ppat.1006527)

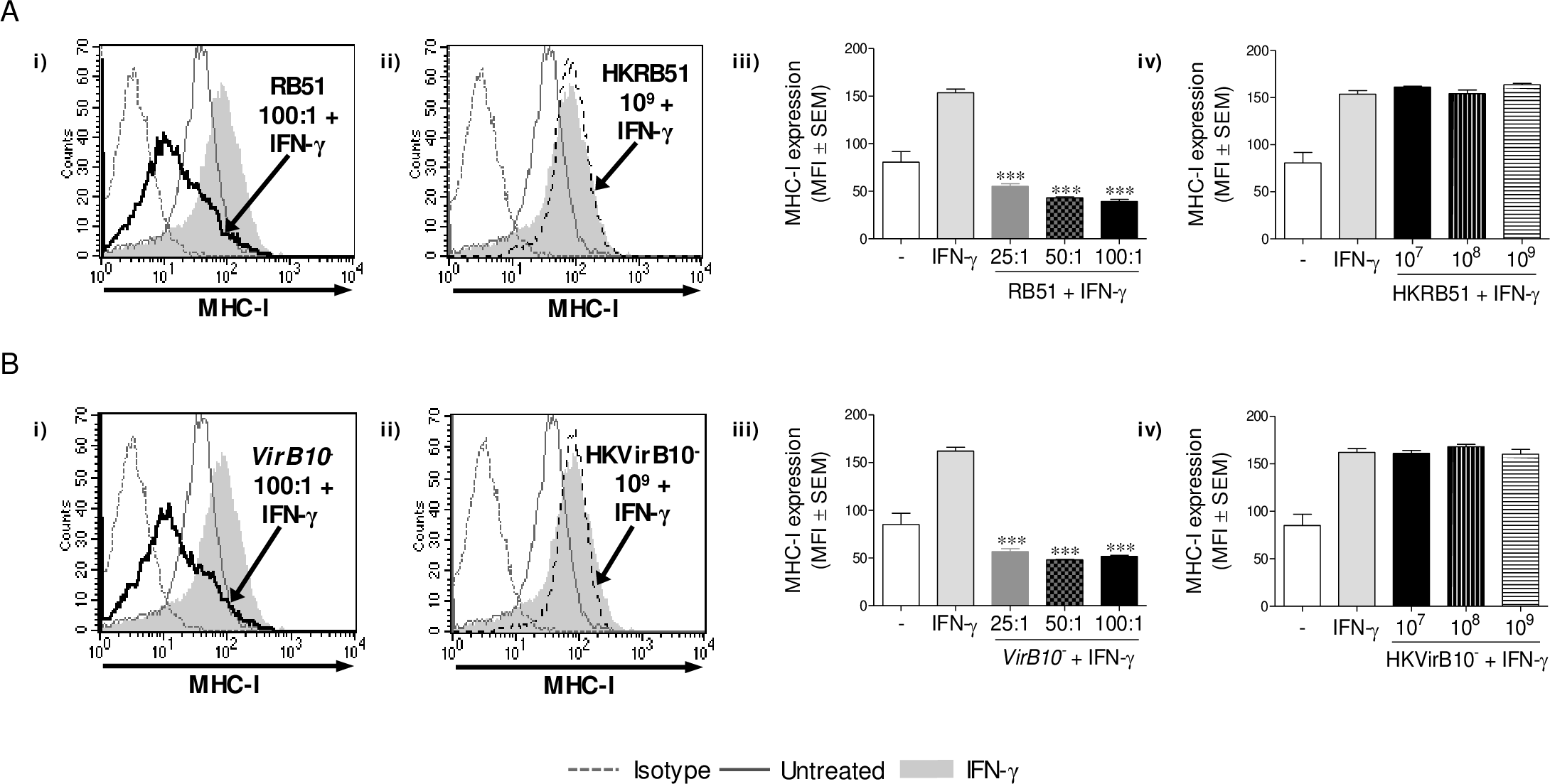

Supplement: S1 Fig — (A and B, Panels i and iii) THP-1 cells were infected with B. abortus RB51 (A) or virB10- (B) at different MOI in the presence of IFN-γ for 2 h, washed and cultured in the presence of IFN-γ for 48 h. (A and B, Panels ii and iv) At the same time, heat-killed bacteria (HK) were used to treat THP-1 cells in the presence of IFN-γ for 48 h. MHC-I expression was assessed by flow cytometry. Bars represent the arithmetic means ± SEM of five experiments. MFI, mean fluorescence intensity. ***P<0.001 vs. IFN-γ-treated. (TIF) [file ppat.1006527.s001.tif]

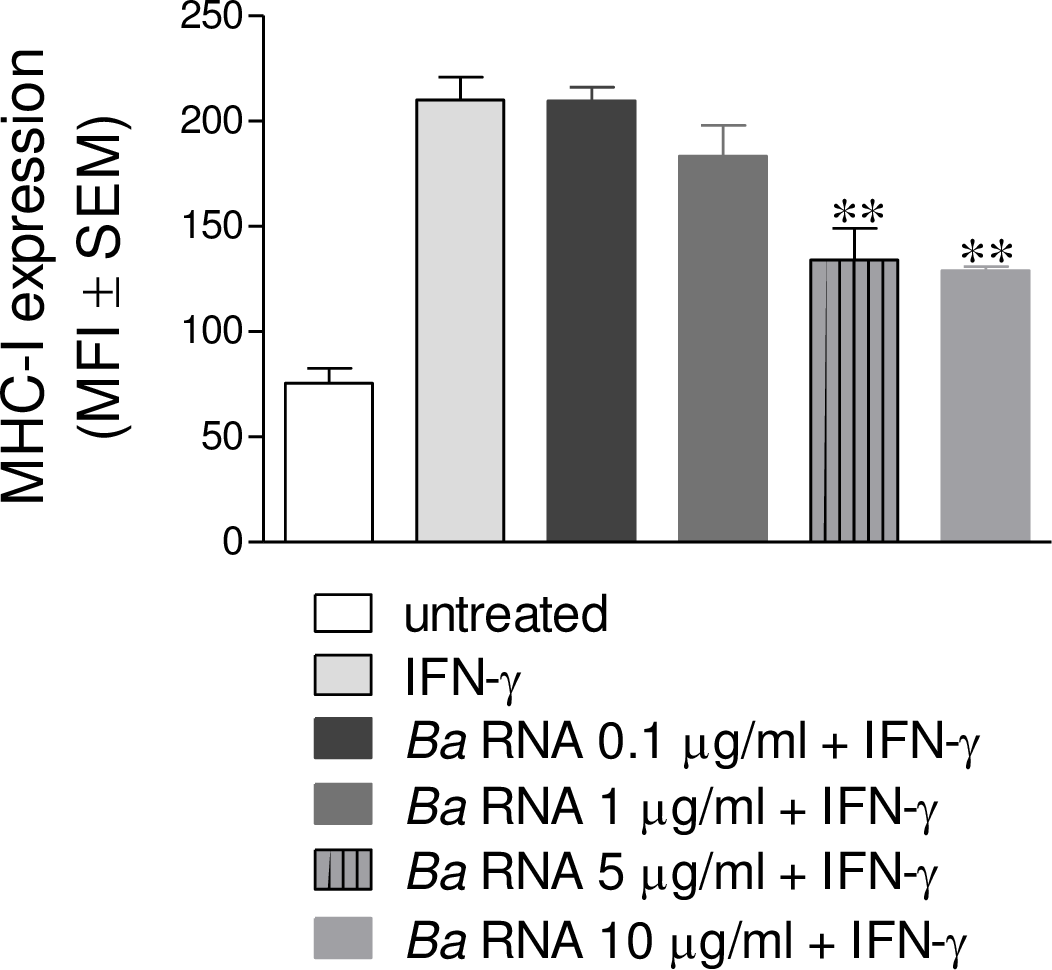

Supplement: S2 Fig — THP-1 cells were treated with different doses of B. abortus RNA purified with Quick-RNA MiniPrep kit in the presence of IFN-γ for 48 h. MHC-I expression was assessed by flow cytometry. Bars indicate the arithmetic means ± SEM of five independent experiments. MFI, mean fluorescence intensity. **P<0.01 vs. IFN-γ-treated. (TIF) [file ppat.1006527.s002.tif]

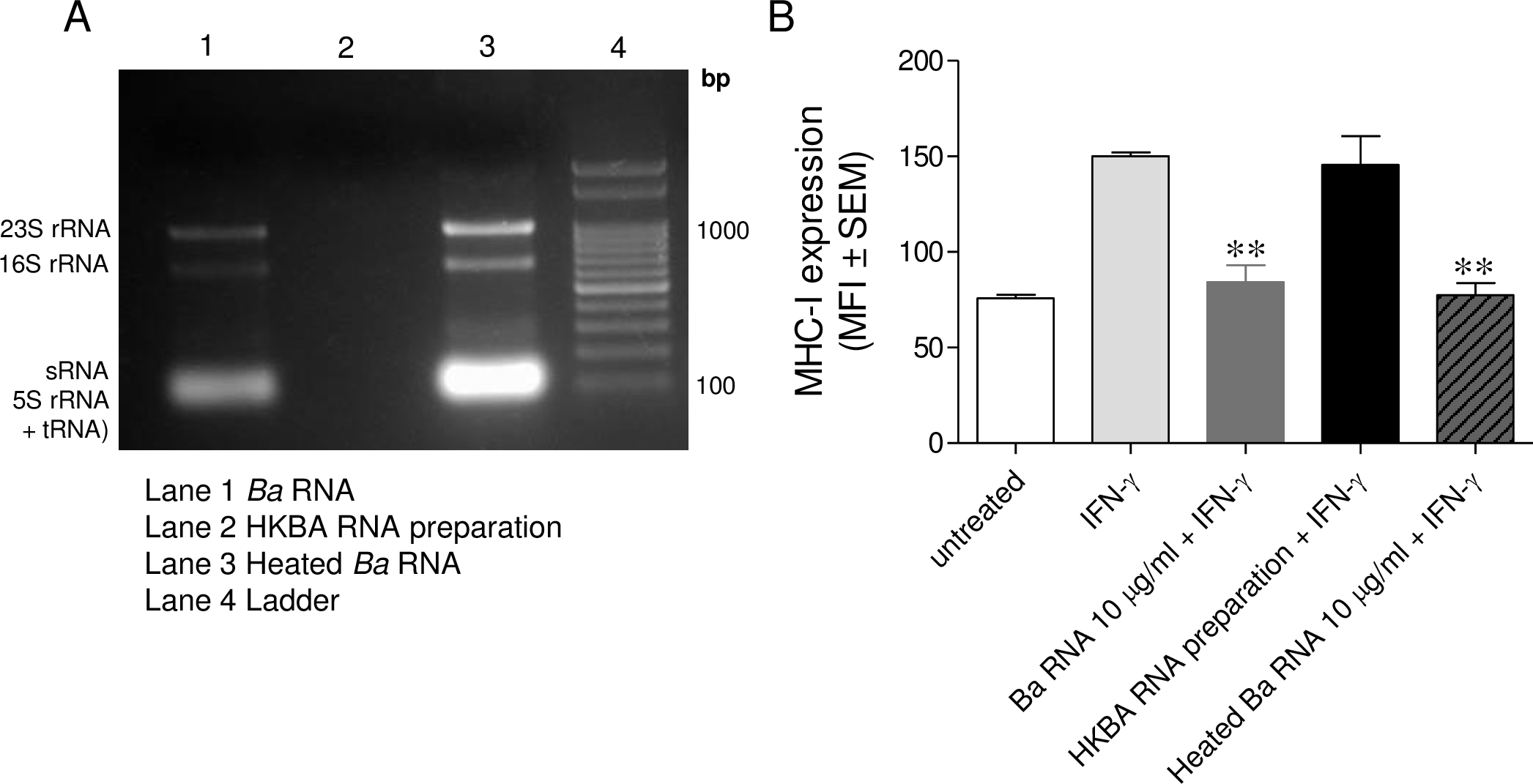

Supplement: S3 Fig — (A) RNA was purified from B. abortus or HKBA. In addition, B. abortus RNA was heat-treated at 70°C for 20 min. Each preparation was visualized by 1% agarose gel electrophoresis. (B) THP-1 cells were stimulated with B. abortus RNA, RNA extraction products from HKBA or heat-treated B. abortus RNA in the presence of IFN-γ for 48 h. MHC-I expression was assessed by flow cytometry. Bars represent the arithmetic means ± SEM of three experiments. MFI, mean fluorescence intensity. **P<0.01 vs. IFN-γ-treated. (TIF) [file ppat.1006527.s003.tif]

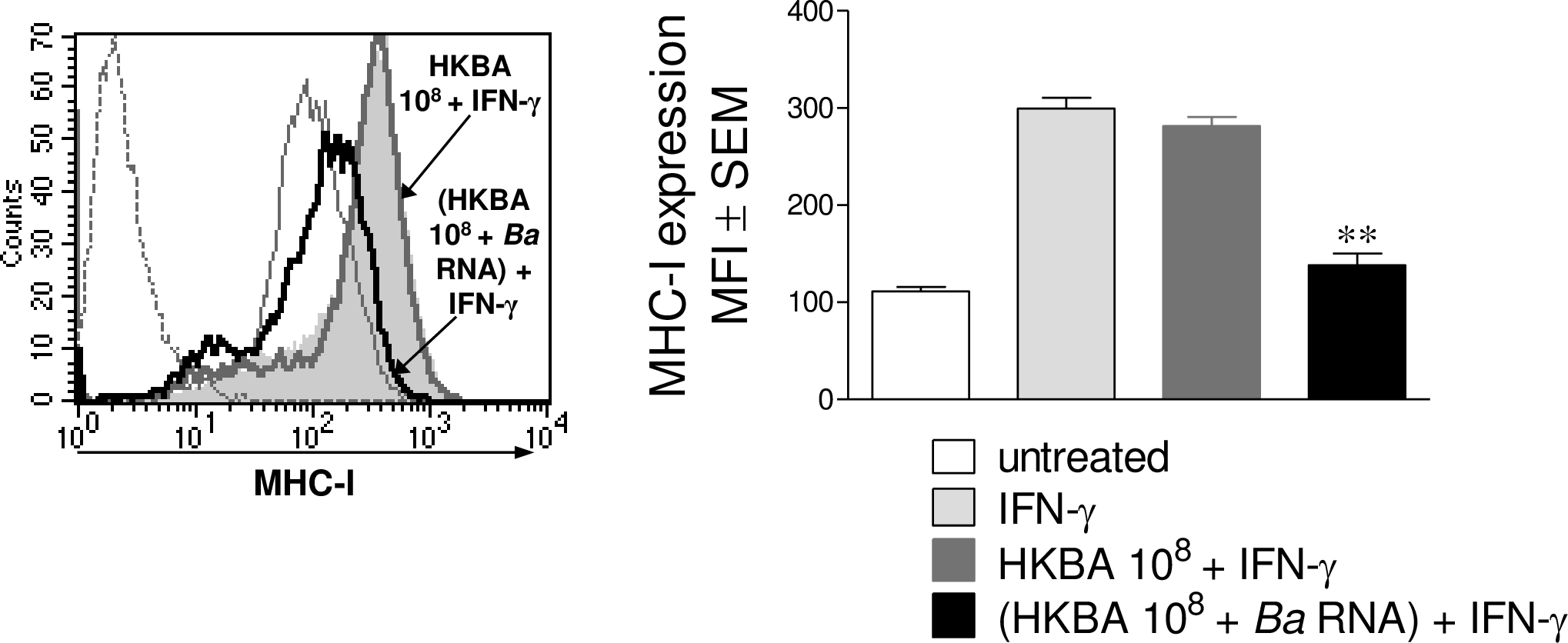

Supplement: S4 Fig — THP-1 cells were treated with HKBA with or without B. abortus RNA in the presence of IFN-γ for 48 h. MHC-I expression was assessed by flow cytometry. Bars indicate the arithmetic means ± SEM of five independent experiments. MFI, mean fluorescence intensity. **P<0.01 vs. IFN-γ-treated. (TIF) [file ppat.1006527.s004.tif]

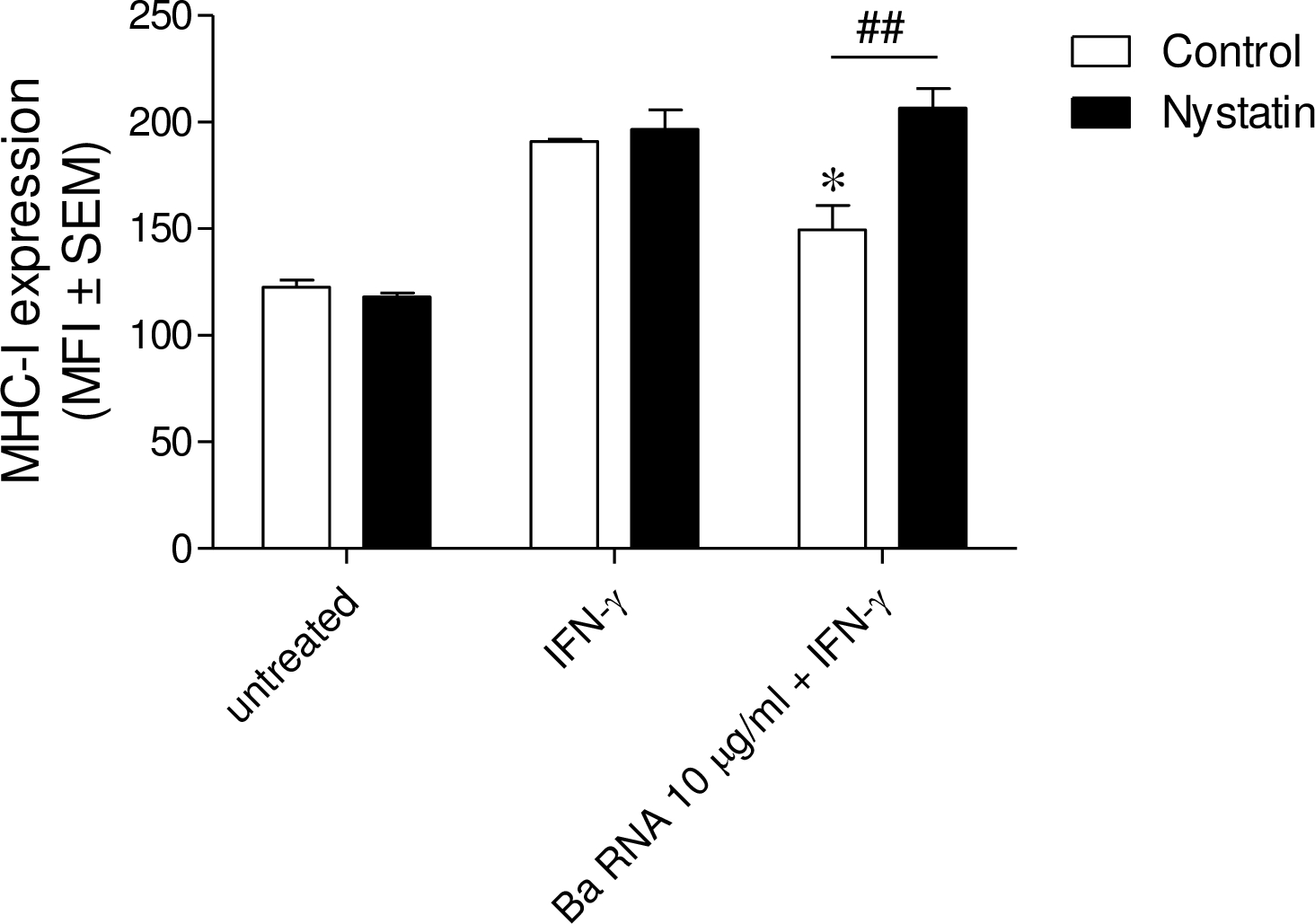

Supplement: S5 Fig — THP-1 cells were treated with B. abortus RNA (10 μg/ml) in the presence of IFN-γ and in the presence or absence of an endocytosis inhibitor (Nystatin) for 48 h. MHC-I expression was assessed by flow cytometry. Bars indicate the arithmetic means ± SEM of five independent experiments. MFI, mean fluorescence intensity. *P<0.05 vs. IFN-γ-treated; ## P<0.01 vs. Control. (TIF) [file ppat.1006527.s005.tif]

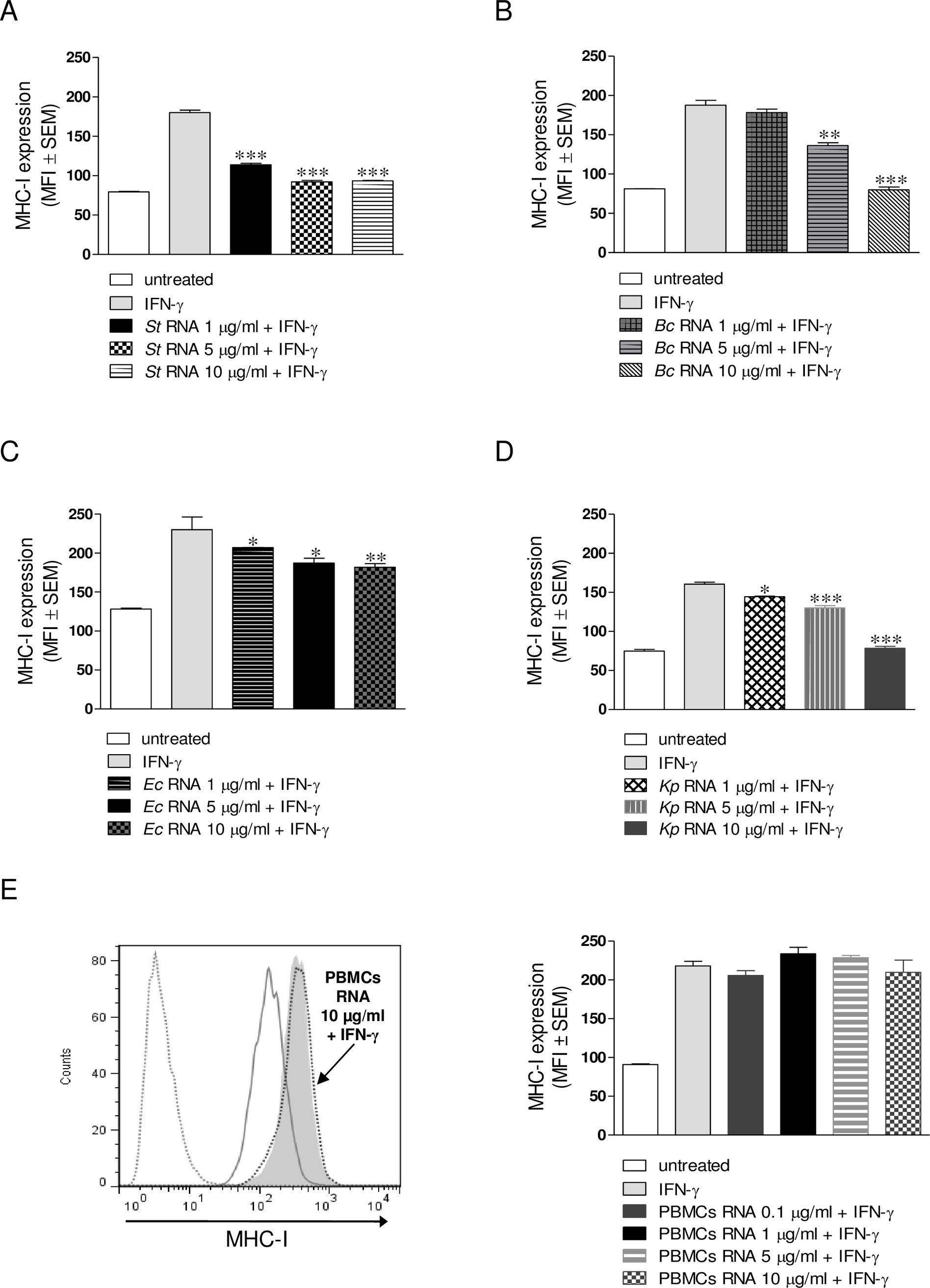

Supplement: S6 Fig — (A-D) THP-1 cells were treated with different doses of S. typhimurium (A), B. cereus (B), E. coli (C) or K. pneumoniae (D) RNAs in the presence of IFN-γ for 48 h. (E) THP-1 cells were treated with different doses of PBMCs RNA in the presence of IFN-γ for 48 h. MHC-I expression was assessed by flow cytometry. Bars indicate the arithmetic means ± SEM of three independent experiments. MFI, mean fluorescence intensity. *P<0.05; **P<0.01; ***P<0.001 vs. IFN-γ-treated. (TIF) [file ppat.1006527.s006.tif]

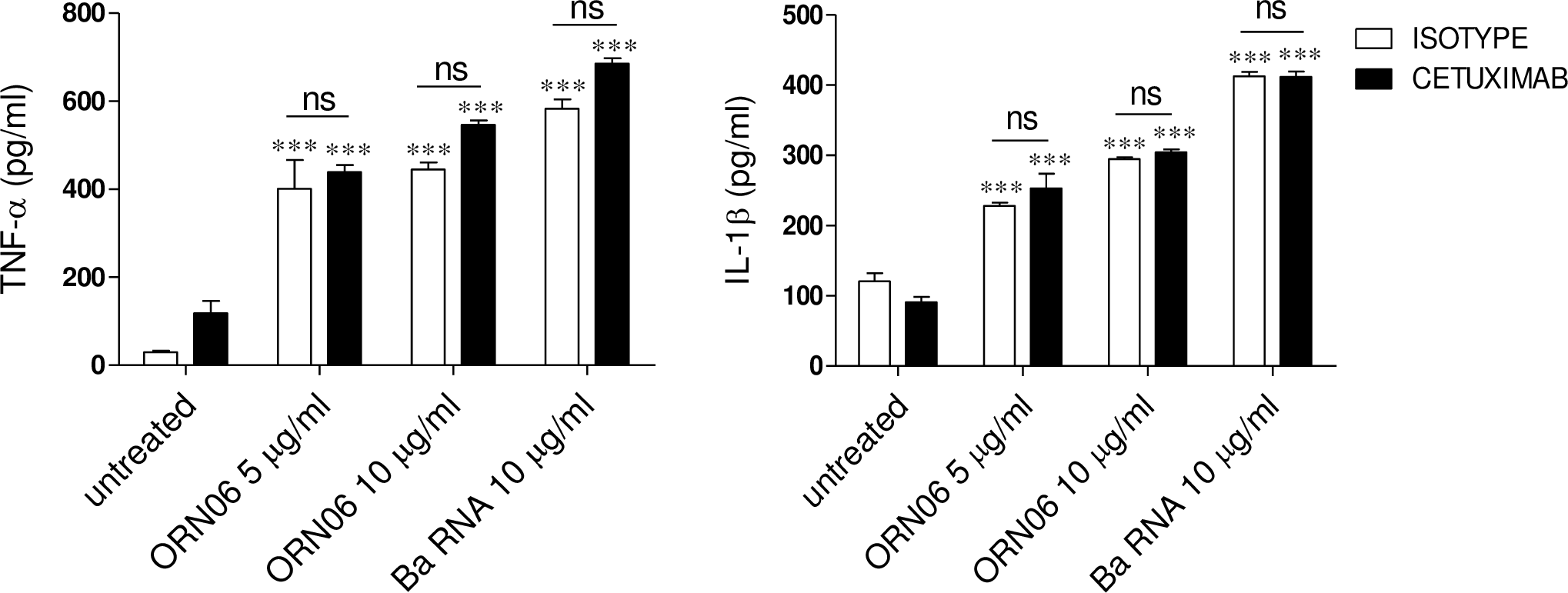

Supplement: S7 Fig — (A and B) THP-1 cells were treated with ORN06 or B. abortus RNA in the presence of Cetuximab or Isotype control for 24 h. Supernatants were then collected and the amount of TNF-α (A) or IL-1β (B) was determined by ELISA. Bars indicate the arithmetic means ± SEM of three independent experiments. ***P<0.001 vs. untreated + Isotype or Cetuximab, accordingly. (TIF) [file ppat.1006527.s007.tif]
